# Cholangiocarcinoma 2020: the next horizon in mechanisms and management

**DOI:** 10.1038/s41575-020-0310-z

**Published:** 2020-06-30

**Authors:** Jesus M. Banales, Jose J. G. Marin, Angela Lamarca, Pedro M. Rodrigues, Shahid A. Khan, Lewis R. Roberts, Vincenzo Cardinale, Guido Carpino, Jesper B. Andersen, Chiara Braconi, Diego F. Calvisi, Maria J. Perugorria, Luca Fabris, Luke Boulter, Rocio I. R. Macias, Eugenio Gaudio, Domenico Alvaro, Sergio A. Gradilone, Mario Strazzabosco, Marco Marzioni, Cédric Coulouarn, Laura Fouassier, Chiara Raggi, Pietro Invernizzi, Joachim C. Mertens, Anja Moncsek, Sumera I. Ilyas, Julie Heimbach, Bas Groot Koerkamp, Jordi Bruix, Alejandro Forner, John Bridgewater, Juan W. Valle, Gregory J. Gores

**Affiliations:** 1 Department of Liver and Gastrointestinal Diseases, Biodonostia Health Research Institute – Donostia University Hospital, University of the Basque Country (UPV/EHU), San Sebastian, Spain; 2 National Institute for the Study of Liver and Gastrointestinal Diseases (CIBERehd, “Instituto de Salud Carlos III”), San Sebastian, Spain; 3 Ikerbasque, Basque Foundation for Science, Bilbao, Spain; 4 Experimental Hepatology and Drug Targeting (HEVEFARM), IBSAL, University of Salamanca, Salamanca, Spain; 5 Department of Medical Oncology, The Christie NHS Foundation Trust, Manchester, UK; 6 Division of Cancer Sciences, University of Manchester, Manchester, UK; 7 Department of Surgery and Cancer, Imperial College London, Hammersmith Hospital, London, UK; 8 Division of Gastroenterology and Hepatology, Mayo Clinic College of Medicine and Science, Rochester, MN USA; 9 Department of Medico-Surgical Sciences and Biotechnologies, Sapienza University of Rome, Rome, Italy; 10 Department of Movement, Human and Health Sciences, Division of Health Sciences, University of Rome “Foro Italico”, Rome, Italy; 11 Biotech Research and Innovation Centre (BRIC), Department of Health and Medical Sciences, University of Copenhagen, Copenhagen, Denmark; 12 Institute of Cancer Sciences, University of Glasgow, Glasgow, UK; 13 Institute of Pathology, University of Regensburg, Regensburg, Germany; 14 Department of Molecular Medicine, University of Padua School of Medicine, Padua, Italy; 15 Digestive Disease Section, Yale University School of Medicine, New Haven, CT USA; 16 MRC-Human Genetics Unit, Institute of Genetics and Molecular Medicine, University of Edinburgh, Edinburgh, UK; 17 Division of Human Anatomy, Department of Anatomical, Histological, Forensic Medicine and Orthopedics Sciences, Sapienza University of Rome, Rome, Italy; 18 Department of Medicine and Medical Specialties, Sapienza University of Rome, Rome, Italy; 19 The Hormel Institute, University of Minnesota, Austin, MN USA; 20 Clinic of Gastroenterology and Hepatology, Universita Politecnica delle Marche, Ancona, Italy; 21 INSERM, Université de Rennes, Rennes, France; 22 Sorbonne Université, INSERM, Centre de Recherche Saint-Antoine (CRSA), Paris, France; 23 Department of Experimental and Clinical Medicine, University of Florence, Florence, Italy; 24 Division of Gastroenterology and Center of Autoimmune Liver Diseases, Department of Medicine and Surgery, San Gerardo Hospital, University of Milano, Bicocca, Italy; 25 Department of Gastroenterology and Hepatology, University Hospital Zurich and University of Zurich, Zürich, Switzerland; 26 Department of Surgery, Mayo Clinic, Rochester, MN USA; 27 Department of Surgery, Erasmus Medical Center, Rotterdam, Netherlands; 28 Barcelona Clinic Liver Cancer (BCLC) group, Liver Unit, Hospital Clínic of Barcelona, Fundació Clínic per a la Recerca Biomédica (FCRB), IDIBAPS, University of Barcelona, Barcelona, Spain; 29 Department of Medical Oncology, UCL Cancer Institute, London, UK

**Keywords:** Biliary tract cancer, Cancer

## Abstract

Cholangiocarcinoma (CCA) includes a cluster of highly heterogeneous biliary malignant tumours that can arise at any point of the biliary tree. Their incidence is increasing globally, currently accounting for ~15% of all primary liver cancers and ~3% of gastrointestinal malignancies. The silent presentation of these tumours combined with their highly aggressive nature and refractoriness to chemotherapy contribute to their alarming mortality, representing ~2% of all cancer-related deaths worldwide yearly. The current diagnosis of CCA by non-invasive approaches is not accurate enough, and histological confirmation is necessary. Furthermore, the high heterogeneity of CCAs at the genomic, epigenetic and molecular levels severely compromises the efficacy of the available therapies. In the past decade, increasing efforts have been made to understand the complexity of these tumours and to develop new diagnostic tools and therapies that might help to improve patient outcomes. In this expert Consensus Statement, which is endorsed by the European Network for the Study of Cholangiocarcinoma, we aim to summarize and critically discuss the latest advances in CCA, mostly focusing on classification, cells of origin, genetic and epigenetic abnormalities, molecular alterations, biomarker discovery and treatments. Furthermore, the horizon of CCA for the next decade from 2020 onwards is highlighted.

## Abstract (web summary)

Cholangiocarcinoma (CCA) comprises heterogeneous biliary malignant tumours, and their incidence is increasing worldwide. This expert Consensus Statement, endorsed by the ENS-CCA, summarizes the latest advances in CCA, including classification, genetics and treatment, and provides recommendations for CCA management and priorities across basic, translational and clinical research.

## Introduction

**Fig. 1:**
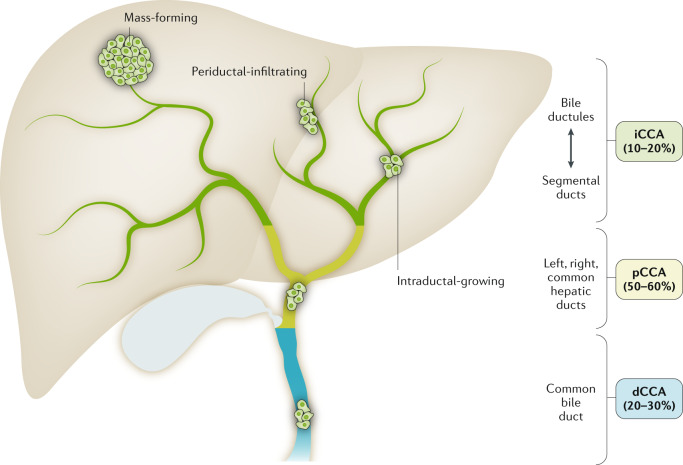
Anatomical classification of cholangiocarcinoma. On the basis of the anatomical site of origin, cholangiocarcinoma (CCA) is classified into intrahepatic CCA (iCCA), perihilar CCA (pCCA) and distal CCA (dCCA). iCCA is defined as a malignancy located in the periphery of the second-order bile ducts, pCCA arises in the right and/or left hepatic duct and/or at their junction, and dCCA involves the common bile duct (that is, the choledochus). Grossly, CCA can show three main patterns of growth: mass-forming, periductal-infiltrating, and intraductal-growing. Mass-forming CCA is a mass lesion in the hepatic parenchyma. Periductal-infiltrating iCCA grows inside the duct wall and spreads longitudinally along the wall. Intraductal-growing CCA is a polypoid or papillary tumour growing towards the duct lumen.

Cholangiocarcinoma (CCA) constitutes a diverse group of malignancies emerging in the biliary tree. CCAs are divided into three subtypes depending on their anatomical site of origin: intrahepatic (iCCA), perihilar (pCCA) and distal (dCCA) CCA^1,2^ (Fig. 1). Of note, considered as an independent entity, mixed HCC–CCA tumours are a rare type of liver malignancy sharing features of both iCCA and HCC and presenting an aggressive disease course and poor prognosis^3,4^. iCCAs arise above the second-order bile ducts, whereas the point of anatomical distinction between pCCA and dCCA is the insertion of the cystic duct. pCCA and dCCA can also be collectively referred to as ‘extrahepatic’ (eCCA)^5^. In the USA, pCCA is the single largest group, accounting for approximately 50–60% of all CCAs, followed by dCCA (20–30%) and iCCA (10–20%)^1,6,7^. CCA is the second most common primary hepatic malignancy after hepatocellular carcinoma (HCC), comprising approximately 15% of all primary liver tumours and 3% of gastrointestinal cancers^1,6,7^. CCAs are usually asymptomatic in early stages and, therefore, often diagnosed when the disease is already in advanced stages, which highly compromises therapeutic options, resulting in a dismal prognosis^1,8^. CCA is a rare cancer, but its incidence (0.3–6 per 100,000 inhabitants per year)^1^ and mortality (1–6 per 100,000 inhabitants per year, globally^9^, not taking into account specific regions with incidence >6 per 100,000 habitants such as South Korea, China and Thailand) have been increasing in the past few decades worldwide, representing a global health problem. Despite advances in CCA awareness, knowledge, diagnosis and therapies, patient prognosis has not improved substantially in the past decade, with 5-year survival (7–20%) and tumour recurrence rates after resection still disappointing^10–17^. Therefore, a detailed study of these types of cancers is urgently needed to improve patient welfare and outcomes. Considering the high heterogeneity of CCAs, individual characterization of these tumours at the genomic, epigenetic and molecular levels is an indispensable approach to ascertain their pathogenesis, paving the path for new therapeutic options and personalized medicine. In this expert Consensus Statement, which is endorsed by the European Network for the Study of Cholangiocarcinoma (ENS-CCA), we provide a comprehensive and critical overview of current knowledge and what is envisaged on the horizon for CCA, focusing on epidemiology, risk factors, clinical presentation, diagnosis, genetic and epigenetic landscape, molecular perturbations, chemoresistance and therapies.

## Methods

This international group of multidisciplinary experts in CCA (that is, oncologists, surgeons, hepatologists, geneticists, immunologists, basic scientists) has been intensively collaborating within the ENS-CCA since 2015 with the main aims of improving our understanding of CCA and the management of patients. In this regard, this expert consensus is endorsed by the ENS-CCA. The overall goal of this multidisciplinary statement is to provide a detailed critical overview of the current knowledge in this field, proposing some expert recommendations and highlighting what is envisaged for the next decade.

**Fig. 2:**
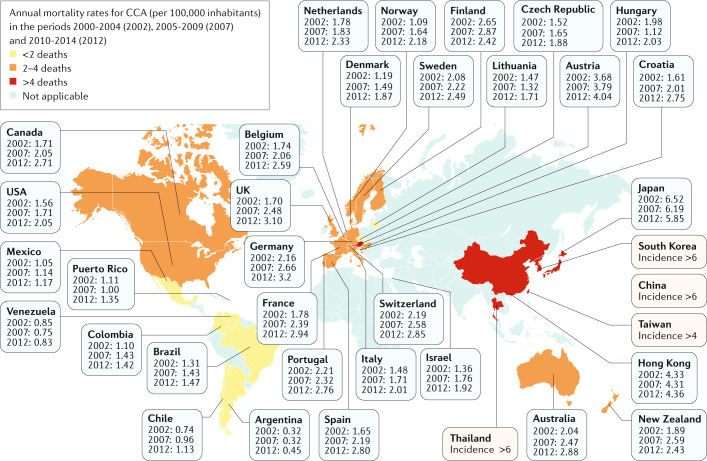
Mortality of cholangiocarcinoma worldwide. Global age-standardized annual mortality rates for cholangiocarcinoma (CCA) (deaths per 100,000 inhabitants, including intrahepatic CCA, perihilar CCA and distal CCA) obtained from Bertuccio et al.^9^. Data refer to the periods 2000–2004 (2002), 2005–2009 (2007) and 2010–2014 (2012). Yellow indicates countries/regions with low mortality (<2 deaths per 100,000 people), orange indicates countries/regions with mortality between 2 and 4 deaths per 100,000 people, and red indicates countries/regions with high mortality (>4 deaths per 100,000 people). Mortality in eastern countries/regions in which CCA is highly prevalent (that is, Thailand, China, Taiwan and South Korea) have not yet been reported and, therefore, CCA incidence is shown for these countries^1^.

J.M.B. and G.J.G. identified the areas of interest, stratified the consensus statement into the sections presented in the document and assigned them to selected ENS-CCA members or non-European collaborators (L.R.R., S.G., S.I.I., J.H. and G.J.G.) who are expert in each field of knowledge and research. To write this document, a PubMed search was conducted by combining the term ‘cholangiocarcinoma’ with the following terms: ‘epidemiology’, ‘risk factors’, ‘classification’, ‘cells of origin’, ‘diagnosis’, ‘staging’, ‘genetics’, ‘epigenetics’, ‘signalling pathways’, ‘epithelial-to-mesenchymal transition’, ‘cancer stem cells’, ‘tumour microenvironment’, ‘immunobiology’, ‘in vitro and in vivo models’, ‘biomarkers’, ‘surgery’, ‘liver transplantation’, ‘therapies’, ‘clinical trials’ and ‘chemoresistance’. No specific search dates were used. All the sections were merged into a first draft by P.M.R and J.M.B. and then extensively revised to create the final document that was later circulated among all the authors for further correction, improvement, discussion and approval. The data presented in Fig. 2 were obtained by combining the values of mortality rates in men and women for both iCCA and eCCA reported in 2019 by Bertuccio et al.^9^. For the recommendations on CCA management and research priorities, ideas were proposed, discussed and approved after final revision by all the authors to reach a consensus.

## Epidemiology and risk factors

The global mortality for CCA increased worldwide during the periods 2000–2004, 2005–2009 and 2010–2014 (Fig. 2), according to the WHO and Pan American Health Organization databases for 32 selected locations in Europe, America, Asia and Oceania^9^. Furthermore, CCA mortality was higher in men than in women worldwide, and in countries/regions in Asia versus those in the West. Accordingly, Asian individuals were reported to have the highest mortality (2.81 per 100,000 men in Japan). However, in the USA, the more noticeable increases in mortality between 2004 and 2014 were found for African American individuals (45%), followed by Asian (22%) and white (20%) individuals^18^. The age-standardized incidence of CCA shows considerable geographical variation, with the highest incidence in Eastern countries/regions; incidence varies from 85 per 100,000 in northeastern Thailand (the highest reported value globally) to 0.4 per 100,000 in Canada^12^. Variations in incidence probably reflect differences in local risk factors and potential genetic predispositions^1,2,19^.

The three subtypes of CCA can have different risk factors, pathobiology, clinical presentations, management and prognosis, as well as distinct epidemiological trends^1,2^. Over the past few decades, the reported age-standardized incidence for iCCA has been steadily increasing in most locations worldwide, whereas the age-standardized incidence for eCCA has been decreasing^2,19^. However, these trends need cautious interpretation given that all versions of the main International Classification of Diseases (ICD) have so far failed to include a separate code for the largest group of CCA (pCCA) and previous versions of ICD–Oncology (ICD-O) have cross-referenced pCCA (technically extrahepatic) to iCCA. Importantly, for the first time, subsequent iterations of both ICD and ICD-O (ICD-11 and ICD-O-4, respectively) — which are due to come into effect by 2021 — will have separate codes for recording iCCA, pCCA and dCCA^20^. Having clearly defined codes for the three subtypes of CCA might facilitate more accurate and meaningful epidemiological data. In the meantime, reported epidemiological trends for CCA and/or biliary tract cancer need to be interpreted carefully.

Furthermore, in the USA, the incidence of iCCA is higher in older people (≥45 years old) than in younger people and in Hispanic individuals than in non-Hispanic individuals, and is associated with a worse 5-year survival in both these populations^21^. Worse overall survival (OS) rates have also been reported for African Americans, followed by American Indians and Alaska Native groups^21,22^. Of note, the hospital charges associated with iCCA management almost doubled from 2005 to 2014 in the USA^22^, and male patients with low annual incomes (<US$37,999) tended to show shorter OS, pinpointing socioeconomic treatment discrepancies that clearly affect outcome^23^. Other factors that affect the interpretation of CCA incidence trends include the unknown effects of improved diagnostics (imaging, endoscopy, histology), greater awareness and the acceptance of performing a biopsy even when imaging is highly suggestive of HCC (in accordance with current guidelines), and increasing the detection of combined HCC–CCA or iCCAs^3,24–28^. Furthermore, there is substantial global variation in rates of microscopically verified (that is, histologically or cytologically confirmed) cases of CCA reported to cancer registries worldwide. For example, Khon Kaen in Thailand, despite being believed to have the highest overall age-standardized incidence of CCA worldwide, has one of the lowest morphologically verified percentages (only 9% of all cases of liver disease)^29^. In this regard, CCA can be notoriously difficult to accurately diagnose due to its location often being inaccessible to histology or cytology, a lack of clear diagnostic imaging criteria, and inaccurate non-invasive tumour biomarkers^1,2^.

Several risk factors, both common and rare, have been linked to CCA (Table 1). Although some risk factors are shared by all forms of CCA, others seem to be more specific for one subtype and seem to be more important in different regions. A common characteristic amongst many of these risk factors is that they are associated with chronic inflammation of the biliary epithelium and bile stasis^19^. Several recognized risk factors have increased globally over recent decades (1990–2016) and could be contributing to increasing CCA rates. For instance, high alcohol consumption, tobacco smoking and viral infections (hepatitis B virus (HBV) and hepatitis C virus (HCV)) have been reported to increase the risk of CCA development^30^. Moreover, it is also important to highlight the global obesity pandemic, as well as the metabolic syndrome and/or presence of nonalcoholic fatty liver disease, as risk factors that deserve future central attention^30^. However, in most locations, the majority of CCA cases remain sporadic, without any identifiable risk factor present. A number of studies are examining the potential influence of commonly used drugs such as aspirin^31–33^ and lipid-lowering statins^34,35^ in the prevention of CCA. Notably, post-diagnosis aspirin usage has been found to be associated with a reduced risk of death (HR 0.71) among patients with CCA^36^. Polymorphisms of host genes encoding enzymes involved in xenobiotic detoxification, DNA repair, multidrug resistance, immune response and folate metabolism have been linked to CCA development^19^. There are currently no published genome-wide association studies (GWAS) in CCA, but an appropriately powered one is eagerly anticipated.

**Table 1:** Risk factors for cholangiocarcinoma

Risk factor	Study type	OR or RR from selected studies
Choledochal cyst^30^	Meta-analysis	OR 26.71 for iCCA OR 34.94 for eCCA
Choledocholithiasis^30^	Meta-analysis	OR 10.08 for iCCA OR 18.58 for eCCA
Cholelithiasis^30^	Meta-analysis	OR 3.38 for iCCA OR 5.92 for eCCA
Cholecystolithiasis^30^	Meta-analysis	OR 1.75 for iCCA OR 2.94 for eCCA
Caroli disease^396^	Population-based study	OR 38 for iCCA OR 97 for eCCA
Primary sclerosing cholangitis^396^	Population-based study	OR 22 for iCCA OR 41 for eCCA
Cirrhosis^30^	Meta-analysis	OR 15.32 for iCCA OR 3.82 for eCCA
Chronic hepatitis B^30^	Meta-analysis	OR 4.57 for iCCA OR 2.11 for eCCA
Chronic hepatitis C^30^	Meta-analysis	OR 4.28 for iCCA OR 1.98 for eCCA
Haemochromatosis^396^	Population-based study	OR 2.1 for iCCA
Inflammatory bowel disease^30^	Meta-analysis	OR 2.68 for iCCA OR 2.37 for eCCA
Chronic pancreatitis^396^	Population-based study	OR 2.7 for iCCA OR 6.6 for eCCA
Liver fluke (*Opisthorchis viverrini*, *Clonorchis sinensis*)^397^	Meta-analysis	OR 5 iCCA > eCCA
Type 2 diabetes mellitus^398^	Meta-analysis	OR 1.73 for iCCA OR 1.5 for eCCA
Nonalcoholic fatty liver disease^399^	Meta-analysis	OR 2.2 for iCCA OR 1.5 for eCCA
Obesity^30^	Meta-analysis	OR 1.14 for iCCA OR 1.2 for eCCA
Hypertension^30^	Meta-analysis	OR 1.10 for iCCA OR 1.21 for eCCA
Alcohol consumption^30^	Meta-analysis	OR 3.15 for iCCA OR 1.75 for eCCA
Cigarette smoking^30^	Meta-analysis	OR 1.25 for iCCA OR 1.69 for eCCA
***Environmental toxins***		
Thorotrast (banned 1969)^400,401^	Retrospective study	RR >300
1,2-Dichloropropane^402^	Retrospective study	RR 15
Asbestos^403^	Case–control study	OR 4.8 for iCCA OR 2.1 for eCCA
Asbestos^404^	Case–control study	OR 1.1–1.7 for iCCA No association with eCCA

eCCA, extrahepatic cholangiocarcinoma; iCCA, intrahepatic cholangiocarcinoma; OR, odds ratio; RR, relative risk.

## Classification and cells of origin

iCCA can emerge at any point of the intrahepatic biliary tree, ranging from bile ductules to the second-order bile ducts (segmental bile ducts). In contrast to HCC, iCCA usually develops in non-cirrhotic liver^37^. pCCA can arise in the right and/or left hepatic duct and/or at their junction (so-called perihilar bile ducts)^38^, and dCCA involves the common bile duct^39^. The current term eCCA is now discouraged as it combines subtypes with distinct clinicopathological features, prognosis and therapeutic options, and also due to the difficulties in discriminating between intrahepatic and extrahepatic origins of pCCA.

**Fig. 3:**
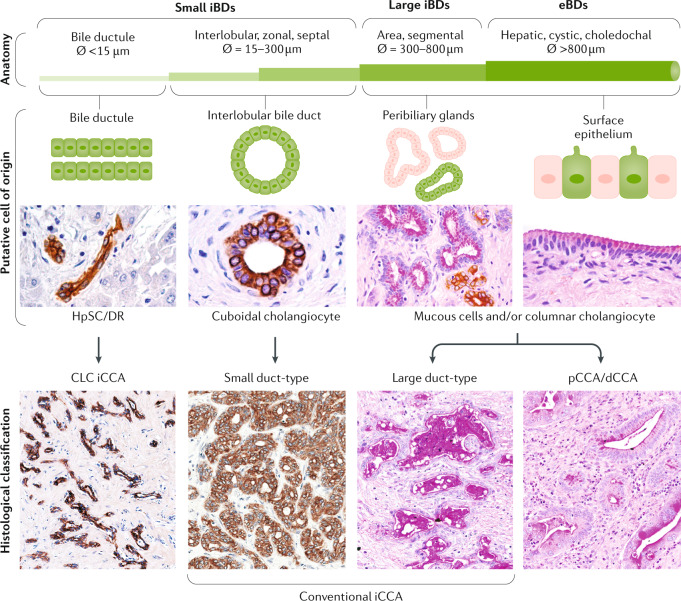
Histological classification and putative cells of origin in cholangiocarcinoma. Based on the duct size, the intrahepatic biliary tree can be further subdivided into small and large intrahepatic bile ducts (iBDs). Small iBDs are lined by small cuboidal cholangiocytes whereas columnar and mucous cholangiocytes line large iBDs. Typically, large iBDs contain peribiliary glands within their wall. The extrahepatic biliary tree shares anatomical features with large iBDs. Histological cholangiocarcinoma (CCA) variants reflect the phenotype of the involved duct and the putative cell of origin. Conventional intrahepatic CCA (iCCA) has two main variants: small duct-type iCCA arises in small iBDs with cuboidal cholangiocytes representing the putative cell of origin, and large duct-type iCCA involves large iBDs and is considered to be derived from columnar cholangiocytes and peribiliary glands (seromucous glands; mucous acini are shown in light pink, serous acini are shown in green). Cholangiolocarcinoma (CLC) is a frequent histological variant of iCCA and its phenotype suggests the origin from bile ductules or ductular reaction (DR) that occurs in chronic liver diseases. The vast majority of perihilar CCA (pCCA) and distal CCA (dCCA) are considered to originate from the lining epithelium and peribiliary glands. This histological subtyping underlies distinct clinicopathological and molecular features as summarized in Table 2. eBD, extrahepatic bile duct; HpSC, human pluripotent stem cell.

iCCA can show three main patterns of growth: mass-forming, periductal-infiltrating, and intraductal-growing^1,38^ (Fig. 1); pCCA and dCCA present as flat or poorly defined nodular sclerosing tumours or, less frequently, as intraductal papillary tumours^40^. CCA can be preceded by pre**-**invasive lesions^39^. Histologically, although the vast majority of pCCA and dCCA are conventional mucin-producing adenocarcinomas or papillary tumours^40^, iCCA shows several histological variants (that is, conventional, cholangiolocarcinoma and rare variants)^41^ (Fig. 3; Table 2). Conventional iCCA can be further classified into two main histological subtypes according to the level or size of the affected duct^42–46^ (Fig. 3; Table 2). Small bile duct iCCA presents as a small-sized tubular or acinar adenocarcinoma with nodular growth invading the liver parenchyma, and with no or minimal mucin production^42–46^. Large bile duct iCCA arises in large intrahepatic bile ducts and comprises mucin-producing columnar tumour cells arranged in a large duct or papillary architecture^38,46–49^. Remarkably, the histological subtyping parallels the high molecular heterogeneity of CCAs and can be ascribed to different cells of origin and pathogenesis^41^. Small bile duct iCCA can be characterized by isocitrate dehydrogenase (*IDH1*, *IDH2)* mutations or fibroblast growth factor receptor 2 (*FGFR2*) fusions^40,50–55^. By contrast, large bile duct iCCA, similar to pCCA and dCCA, shows a high frequency of mutations in *KRAS* and/or *TP53* genes^51,53,56,57^. Interestingly, dCCA is also associated with *ELF3* mutations^58^. Growing evidence demonstrates that distinct cells of origin within an organ can give rise to different subtypes of cancer, typically tissue-specific stem and progenitor cells^59–62^. Evidence regarding the cells of origin of CCA in humans was obtained by phenotyping the candidate tissues and/or cells of origin with respect to CCA subtypes through histological and gene expression analysis^38,44,46,63–68^, whereas indirect evidence might be derived from risk factors^67,68^.

**Table 2:** Clinicopathological and molecular features of cholangiocarcinoma

CCA type	Gross pattern	Precancerous lesion	Underlying disease	Tissue markers^a^	Frequent mutations
iCCA — CLC	Mass-forming	None	Viral, cirrhosis	NCAM	*IDH1/2*, *FGFR2* fusions, *BAP1*, *BRAF*, *ARID1A*, *KRAS*, *TP53*, *SMAD4* Increased *IDH1* and *TP53*
iCCA — small duct type	Mass-forming	None	Viral, cirrhosis	NCAM, N-cadherin, SMAD4, BAP1^loss^	*IDH1/2*, *FGFR2* fusions, *BAP1*, *BRAF*, *ARID1A*, *KRAS*, *TP53*, *SMAD4* Increased *IDH1*/*2*, *FGFR2* fusion
iCCA — large duct type	Periductal infiltrating (±mass-forming) or intraductal growing	Biliary epithelial neoplasia, IPNB, ITPN, mucinous cystic neoplasm	Primary sclerosing cholangitis, liver flukes	Mucin^b^, MUC5AC, MUC6, S100P, SMAD4^loss^, BAP1	*IDH1/2*, *FGFR2* fusions, *BAP1*, *BRAF*, *ARID1A*, *KRAS*, *TP53*, *SMAD4* Increased *KRAS* and *TP53*
pCCA–dCCA	Periductal infiltrating or intraductal growing	Biliary epithelial neoplasia, IPNB, ITPN, mucinous cystic neoplasm	Primary sclerosing cholangitis, liver flukes	Mucin^b^, MUC5AC, MUC6, S100P, SMAD4^loss^, BAP1	*KRAS*, *TP53*, *SMAD4*, *ERBB3*, *PRKACA*–*PRKACB* fusions, *ELF3*

CCA, cholangiocarcinoma; CLC, cholangiolocarcinoma; dCCA, distal cholangiocarcinoma; iCCA, intrahepatic cholangiocarcinoma; IPNB, intraductal papillary neoplasm of the bile duct; ITPN, intraductal tubulopapillary neoplasm; pCCA, perihilar cholangiocarcinoma. ^a^Markers from single-centre experience; international criteria and consensus on a definite panel of markers are still needed. ^b^Mucin refers to histomorphological stains periodic acid–Schiff (PAS) or Alcian PAS.

Small bile duct and cholangiolocarcinoma iCCA subtypes emerge at the level of smaller intrahepatic bile ducts, including bile ductules^38,44,46,69^. In these portions of the biliary tree, hepatic stem or progenitor cells (HpSCs) and cuboidal cholangiocytes represent surface epithelium and are the putative cells of origin of these malignancies^38,44,46,69^ (Fig. 3; Table 2). Interestingly, HpSCs have been implicated in CK19^+^ HCC^70^ and in combined HCC–CCA^4,64,71^. Notably, CCA-like HCC tumours display embryonic stem cell-like expression traits, further substantiating the involvement of bipotent hepatic progenitor cells, and in humans display a worse prognosis than HCC^72^. In line with these findings, nestin, a maker of bipotent progenitor oval cells, is greatly increased in HCC–CCA tumours and is associated with a worse prognosis, and has been proposed as a new possible diagnostic and prognostic biomarker^3,26^. Small bile duct and cholangiolocarcinoma iCCA usually develop on a background of chronic liver disease (such as chronic viral hepatitis and cirrhosis)^38,46,70^, characterized by HpSC activation^71,73,74^.

Large bile duct iCCA, pCCA and dCCA derive from columnar mucous cholangiocytes or peribiliary glands^38,44,46,47,49,69^ (Fig. 3), which are also implicated in the origin of precursor lesions (such as intraductal papillary neoplasm)^46^. These malignancies mainly develop in ducts affected by chronic inflammation as in primary sclerosing cholangitis (PSC) or liver fluke infection^46,49,69^. In PSC, peribiliary gland cell proliferation, mucinous metaplasia, and dysplasia to cancer progression take place within bile ducts and along the biliary tree, mimicking the cancerization field (‘field defect’)^49,75^.

Controversies exist regarding the cellular origins of iCCA based on lineage tracing studies in experimental carcinogenetic models^76^. Indeed, there is evidence in favour of HpSC, cholangiocyte or hepatocyte origin of iCCA from these experimental settings^76–81^. Thus, a definitive determination of the origin of iCCA in humans cannot be reached based on current evidence and requires further research. Moreover, it should be underlined that current experimental models of liver damage do not fully recapitulate the pathogenesis of human chronic liver disease, including proliferative senescence in hepatocytes^66,82,83^, and that lineage tracing studies must be conducted and interpreted cautiously^76,84–87^.

## Clinical presentation

### Diagnosis

CCAs are usually asymptomatic during early stages. The most frequent symptom of pCCA and dCCA is jaundice due to biliary tract obstruction^88^. In iCCA, jaundice is less frequent and mostly associated with advanced disease. Other symptoms of advanced disease include asthenia, abdominal pain, malaise, nausea, anorexia and weight loss. iCCA is an incidental finding in around 20–25% of cases^88^. In patients with cirrhosis, ultrasonography surveillance for HCC enables iCCA diagnosis at an asymptomatic, early stage^89^. Unfortunately, the majority of iCCA cases occur in the absence of known risk factors^90^, when the only chance for early diagnosis is by cross-sectional imaging performed for other reasons.

Imaging techniques, such as ultrasonography, contrast-enhanced ultrasonography (CEUS), CT and MRI, play a key part in the management of CCA in terms of diagnosis, staging, follow-up and assessment of treatment response. Their diagnostic accuracy is influenced by the anatomical location and growth patterns of CCA, and their use for staging varies according to tumour location^91^. CT is considered the standard imaging method for the preoperative assessment of both iCCA and pCCA; it provides a comprehensive evaluation of the primary tumour, the relationship with adjacent structures, and potential thoracic and abdominal spread^91^. MRI has similar accuracy to CT for diagnosis and staging, but it incorporates specific sequences such as diffusion-weighted imaging and the potential for performing magnetic resonance cholangiopancreatography (MRCP), which is critical for pCCA staging^92^. The most frequent imaging patterns displayed by iCCA on both CT and MRI are arterial peripheral rim enhancement with progressive homogeneous contrast agent uptake until the delayed or stable uptake during late dynamic phases^93,94^. A targetoid pattern defined as arterial rim enhancement, peripheral washout and delayed central enhancement can also be present in iCCA^95^. When gadoxetic acid is used, the washout should be read in the portal phase instead of in delayed phases to prevent misclassification between HCC and iCCA in a cirrhotic liver^96^. More controversial is the use of CEUS in iCCA, particularly in the setting of underlying chronic liver disease. iCCA exhibits homogeneous arterial hyperenhancement followed by venous washout in near 50% of patients, a pattern indistinguishable from that found in HCC^94,97^. However, in a relevant proportion of patients with iCCA, washout takes place earlier than 60 s after contrast agent injection; this feature is rarely observed in HCC, and the intensity of washout in the portal phase is more marked in iCCA than in HCC^89^. These refinements might decrease the risk of misdiagnosis in HCC^98^, and have been adopted by the Liver Imaging Reporting Data System (LI-RADS) for CEUS (LI-RADS-CEUS)^99^. No evidence supports the use of ^18^F-FDG PET for completion of staging, which could be of special value to exclude the presence of lymph node or distant metastases^100^.

As no specific CCA radiology pattern exists, histopathological or cytological analysis is mandatory to confirm the diagnosis^1,28^. This diagnosis is based on the WHO classification of biliary tract cancer showing an adenocarcinoma or mucinous carcinoma^101^, with tubular and/or papillary structures and a variable fibrous stroma^102^.

### Staging

There is no widely used staging system for CCA, although it can be staged according to the American Joint Committee on Cancer (AJCC) TNM system^103,104^. Despite providing a clinically meaningful classification correlated with prognosis^105^, the current TNM classification has some limitations. First, it has limited discriminatory ability between T2 and T3 tumours in surgically resected iCCAs^105,106^. T2 tumours include multifocal disease or disease with intrahepatic vascular invasion that probably reflect disseminated disease and the OS in patients with these tumours does not differ from the OS in patients with extrahepatic metastatic disease^105^. Similarly, there is also evidence supporting the negative effect of the presence of multifocal iCCA (iCCA with liver metastases; T2) on prognosis (OS) when compared with other early stages, which might require consideration in future versions of the AJCC TNM classifications^107^. Second, although size has been included for the first time as a prognostic factor for iCCA in the eighth edition of the *AJCC Cancer Staging Manual*, the only cut-off size considered is 5 cm in T1 tumours. Several authors have shown that a 2 cm cut-off value might identify very early tumours with very low likelihood of dissemination and potential long-term survival with low recurrence rates^24,108^. Finally, the TNM classification misses relevant prognostic factors such as the presence of cancer-related symptoms (such as abdominal pain or malaise) or the degree of liver function impairment. As previously shown with HCC, future proposals from society guidelines should focus on stratifying non-surgical patients for clinical studies using clinical and imaging data. Notably, Chaiteerakij et al. proposed a new staging system for pCCA based on tumour size and number, vascular encasement, lymph node and peritoneal metastasis, Eastern Cooperative Oncology Group (ECOG) performance status (ECOG-PS), and CA19-9 level, which has shown a better performance in predicting survival than the TNM staging system^109^. Also, important for stratification in clinical trials, radiographic staging parameters need to be developed in the absence of histological staging, and a radiographic staging system has been proposed for pCCA^109^.

## Genetics and epigenetics

### Genomics

Initial efforts using integrative genomics approaches to stratify CCA based on prognosis have highlighted extensive deregulated transcriptomic landscapes showing augmented anti-apoptotic signalling, angiogenesis, signal transduction and transcriptional control^8,110^. The main oncogenic networks comprised WNT-CTNNB1, MYC, ERBB, TNF and VEGF signalling, emphasizing cell survival signalling pathways in patients with poor OS^8^. Regarding genomic alterations, CCA falls midway in the mutational spectrum of cancers^111^, with an approximately equal content of genomic alterations in iCCA (median 39 non-synonymous mutations per tumour) and eCCA (median 35 non-synonymous mutations per tumour)^56^. Massive sequencing studies^56,112–121^ have improved our understanding of the causal mechanisms in CCA, emphasizing the genomic complexity in prevalent oncogenic modules affecting: cell cycle regulation; DNA damage and genomic instability (*TP53*, *CDKN2A*, *CCND1*, *ATM*, *ROBO2*, *BRCA1* and *BRAC2*); MYC amplification; epigenetic regulation including NADPH metabolism (*IDH1* and *IDH2*), de-ubiquitination (*BAP1*), SWI–SNF complex (*PBRM1*, *ARID1A*, *ARID1B*, *ARID2*, *SMARCA2*, *SMARCA4* and *SMARCAD1*) and histone (de-)methylation (*MLL2*, *MML3*, *KMT2C*, *KDM4A*, *KDM5D*, *KDM6A* and *KDM6B*); kinase signalling (*KRAS*, *ERBB1–3*, *BRAF*, *PIK3CA*, *PTEN*, *STK11*, *SMAD4* and *FGFR1–3*); immune dysregulation (JAK–STAT3 signalling); *FGFR2* and *PRKCA–PRKCB* fusions; the WNT–CTNNB1 pathway (*APC*); Hippo signalling (*NF2*, *SAV1* deletion); *METLL13* amplifications; and deregulated Notch signalling. Interestingly, the predominant genomic alterations in CCA are associated with epigenetic processes^122^. Indeed, the most clinically significant genomic breakthroughs in iCCA are the discovery of hotspot *IDH* mutations (*IDH1*^*R132*^ and *IDH2*^*R172*^) that cause an accumulation of the oncometabolite 2-hydroxyglutarate (2-HG)^57^, as well as the constitutive active gene fusion event between *FGFR2* and many different partners, including the most prevalent (*BICC1* (refs^50,112–114^), *PPHLN1* (ref.^115^), *TACC3* (ref.^112^) and *MGEA5* (ref.^112^)). These alterations are important as they are driving current marker-based phase III clinical trials testing specific agents targeting these alterations in *FGFR2* fusion-positive CCA (NCT03773302)^123,124^ and *IDH*-mutated CCA (NCT02989857).

To date, information on the inherited predisposing genetic risk factors causing CCA is very limited^125^. Data mostly stem from GWAS of patient cohorts diagnosed with PSC^25,126^, with increased risk of CCA. However, the only detailed genomic association with aetiological risk factors investigated by genome sequencing has been the association with liver fluke infection (*Opisthorchis viverrini* and *Clonorchis sinensis*), with fluke-positive tumours showing an overall higher mutational rate (median 4,700 versus 3,143 somatic mutations per tumour)^116^ with prevalent mutations in *SMAD4* and *TP53* as well as *ERBB2* amplifications^116–118^. Furthermore, although not in a high proportion, *KRAS* mutations have been recurrently found in all CCA subtypes^56,116,117^. A statistically significant association has also been observed between *TP53* mutation and HBV infection^119,120^. Few studies have investigated the molecular distinction between iCCA, pCCA and dCCA^8,56,116,121^. Nakamura et al. emphasized the difference in anatomical location of the tumour, highlighting *IDH*, *EPHA2* and *BAP1* mutations and *FGFR2* fusions in iCCA, whereas extrahepatic tumours specifically show *PRKACA* and *PRKACB* fusions as well as mutations in *ELF3* (similar to tumours in the ampulla of Vater)^127^ and *ARID1B*^56^. Based on these fundamental causal alterations, tumours in distinct anatomical sites should probably be treated differently. Besides linking *IDH* mutations with the response to ivosidenib^128^, few studies have related genomic alterations to high-throughput drug screening^119,129,130^. Among these, Nepal et al. used an approach of integrative genomics in a large cohort of iCCAs to elucidate unique mutational signatures, structural variants and epigenomic alterations, emphasizing specific oncogenetic mechanisms in four distinct subsets of patients with potential drug responses and categories: RNA synthesis inhibition, *IDH* mutant; microtubule modulator, *KRAS* mutant; topoisomerase inhibition, *TP53* mutant; and mTOR inhibitors^119^.

### Epigenetics

Epigenetics was shown to play an important part in the initiation and progression of CCA, affecting tumour phenotype in the absence of changes in DNA sequences^131^. Deregulated patterns of methylation, histone modifications and aberrant expression of non-coding RNAs promote unbalanced transcription and gene expression that impair cell homeostasis and sustain malignant transformation. Growing evidence supports deregulated methylation motifs in CCA cells compared with their normal counterparts, with a prevalent hypermethylation of multiple CpG sites occurring in CCA^132,133^. One of the largest studies of integrative genetic and epigenetic analyses in CCA, including 489 CCAs from ten countries/regions, has shown how the molecular make-up of CCA goes beyond the differentiation according to anatomical site^116^. Indeed, by combining DNA sequencing with transcriptomic and DNA methylation analyses, four clusters of CCA with different clinical outcomes were identified. Two sets of hypermethylated CCAs stood out, with an interesting association between CpG island hypermethylation and liver fluke-related tumours, increased mutation rate, downregulation of the DNA demethylation enzyme TET1, upregulation of the histone methyltransferase EZH2 and an increased level of deamination events. Conversely, the subgroup of iCCAs with enrichment in *IDH1/2* and *BAP1* mutations, as well as *FGFR* translocations, showed hypermethylation of the CpG shores (the regions immediately flanking CpG islands, up to 2 kb away). This different pattern suggests how early epigenetic deregulation caused by external carcinogenic events (for example, liver flukes) are at the basis of CCA development in the first cluster, whereas in the second cluster, epigenetic aberrations probably arise as a downstream consequence of somatic mutations (*IDH*) that produce oncometabolites responsible for the DNA hypermethylation. These differences have remarkable clinical implications, because on the one hand early epigenetic events might be used for early detection of tumours in the first cluster (by using quantitative DNA methylation markers in the bile of individuals at risk)^80^ and on the other hand, the tumour clonal mutations might be a marker of effective targeted therapies (such as IDH inhibitors).

**Fig. 4:**
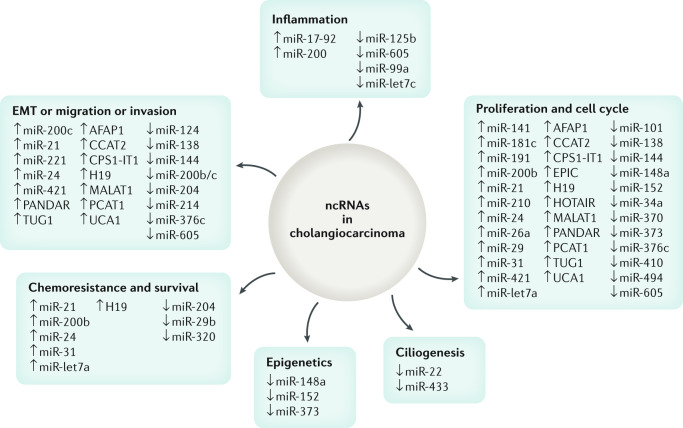
Non-coding RNAs in cholangiocarcinoma and their relationship with different tumorigenic processes. Non-coding RNAs (ncRNAs) that have been found to be dysregulated (up or down) in cholangiocarcinoma and that have key roles in the regulation of cellular processes, such as proliferation, cell cycle, ciliogenesis, epigenetics, inflammation, chemoresistance, survival, epithelial to mesenchymal transition (EMT), migration and invasion are shown.

Methylome data can also provide insights into the cells of origin of CCA. Tumours with high genetic and epigenetic occurrence seem to have an enrichment of events within embryonic stem cell-related bivalent regulation^134–136^. *IDH*-mutated tumours instead seem to resemble the profile of cholangiocellular CCAs that show gene expression traits of epithelial–mesenchymal transition (EMT)^136^. Histone modifications have been less studied in CCA. Histone deacetylase (HDAC) enzymes are responsible for regulation of histone acetylation that ultimately affects chromatin organization. HDAC were found to be upregulated in CCA in vitro^137^, and are being investigated as targets of treatment. Evidence also suggests that HDAC inhibitors, as well as dasatinib, might be particularly active in *IDH*-mutated tumour cells^129,130^. Non-coding RNAs account for around 98% human RNAs and include microRNAs (miRNAs) and long non-coding RNAs, among others. These non-coding RNAs regulate the expression of a plethora of target genes affecting all the hallmarks of the cancer phenotype from cell proliferation and migration to EMT and the regulation of the primary cilium in cholangiocytes^138–142^ (Fig. 4).

## Signalling and molecular networks

CCA often arises in the setting of prolonged biliary inflammation and/or cholestasis, which contribute to carcinogenesis. According to transcriptomic profiles, the ‘inflammation’ (38%) and ‘proliferation’ (62%) subtypes of iCCA were previously identified and reported to be differentially enriched with activation of the pro-inflammatory and oncogenic pathways, respectively^110^. The inflammation subclass of tumours was characterized by induction of immune-related signalling pathways. By contrast, the proliferation subclass was enriched in classic oncogenic pathways, including deregulated receptor tyrosine kinase (RTK) signalling, RAS–RAF–ERK, PI3K–AKT–mTOR, insulin growth factor receptor 1, MET, polo-like kinase 1, aurora kinase A, *KRAS* mutations and stem-like genomic traits as well as a focal deletion in the Hippo pathway (*SAV1*)^8,110,143^. Notably, patients with the proliferation subtype of iCCA displayed decreased OS (median 24.3 months versus 47.2 months for those with the inflammation subtype; *P = *0.048).

**Fig. 5:**
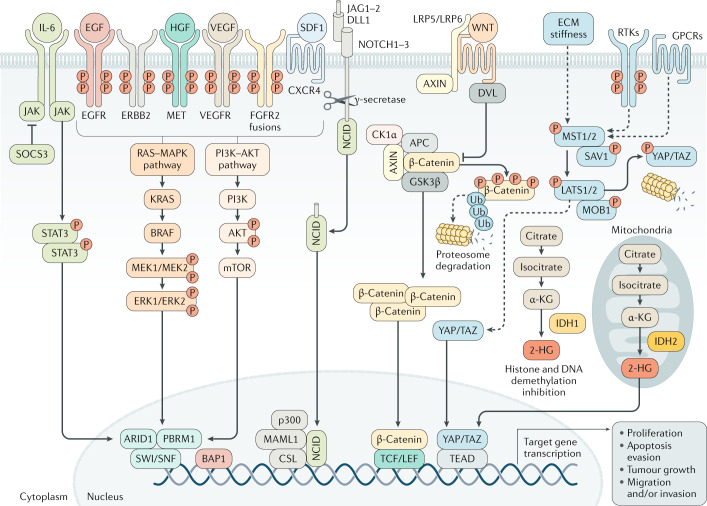
Signalling pathways involved in cholangiocarcinoma development and progression. The process of cholangiocarcinogenesis, and further tumour evolution and growth, involves complex and heterogeneous processes that include the interplay of extracellular ligands (such as pro-inflammatory cytokines, growth factors and bile acids, among others), which are present in the tumour microenvironment, and increased expression and/or aberrant activation of cell surface receptors and the deregulation of intracellular signalling pathways, finally leading to cell proliferation, survival and migration or invasion. The most common genes that might be mutated or amplified resulting in the overactivation of some of these pathways are *KRAS*, *BRAF*, *ARID1*, *PBRM1*, *BAP1*, *IDH1* and *IDH2*. The activation of these signalling pathways might also occur as a result of the interaction between the tumour epithelia and the tumour reactive stroma. 2-HG, 2-hydroxyglutarate; ECM, extracellular matrix; RTK, receptor tyrosine kinase.

Cholangiocarcinogenesis is orchestrated by a complex interplay of extracellular ligands (such as pro-inflammatory cytokines, growth factors and bile acids, among others), which are present in the tumour microenvironment (TME), and increased expression and/or aberrant activation of cell surface receptors and the deregulation of intracellular signalling pathways, finally leading to cell proliferation, survival and genetic and/or epigenetic alterations (Fig. 5).

Chronic inflammation and fibrosis facilitate cholangiocyte transformation in a multistep manner, providing extracellular ligands that modulate several signalling pathways. In particular, sustained IL-6–STAT3 signalling was shown to contribute to mitogenesis by upregulating myeloid cell leukaemia 1 (MCL1) or altering *EGFR* promoter methylation^144,145^. Similarly, bile acids are not genotoxic but might also promote cholangiocarcinogenesis through a mechanism involving the activation of EGFR, induction of COX2, MCL1 and IL-6, and downregulation of farnesoid X receptor (FXR)^146,147^. Of note, FXR expression was reported to be decreased in human CCA tumours compared with surrounding normal liver tissue, correlating with tumour differentiation^140^. By contrast, the levels of TGR5, another bile acid receptor, were found to be increased in CCA tumours and to be correlated with a worse prognosis (perineural invasion)^140^. CCA tumours, and particularly iCCAs and pCCAs, are characterized by a reactive desmoplastic stroma containing cancer-associated fibroblasts (CAFs) that crosstalk with CCA cells secreting paracrine factors such as heparin-binding EGF-like growth factor, stromal-cell derived factor 1 (SDF1), platelet-derived growth factor (PDGF)-B and extracellular matrix (ECM) proteins^148^.

Although there are marked differences in the genomic features depending on the anatomical location and risk factors, activation of the RTK signalling pathway is a common event in CCA across subtypes. In this regard, aberrant *EGFR*, *ERBB2* and *MET* RTK expression has been found in different CCA subclasses that are associated with worse prognosis^8,110^. RTK signalling mainly triggers the activation of the RAS–MAPK and PI3K–AKT–mTOR pathways. Furthermore, RAS–MAPK pathway activation due to *KRAS*-activating mutations is found in all CCAs without distinction, whereas *BRAF* mutations are more prevalent in iCCA^149^. Interestingly, chromosomal oncogenic gene fusion rearrangements involving FGFR2 RTK occur almost exclusively in iCCA^50,52,56,112,113^. Besides *FGFR2* fusions, ROS1 kinase protein fusions have also been identified in iCCA^150^. Thus, RTK signalling pathways present actionable molecular alterations that are amenable for therapeutic targeting at multiple levels. *IDH1* and *IDH2* encode metabolic enzymes that interconvert isocitrate and α-ketoglutarate^51,53,113,117,151^. Mutations in *IDH1* and *IDH2* lead to the production of high levels of 2-hydroxyglutarate, an oncometabolite that interferes with histone and DNA demethylases and inhibits the mitochondrial electron transport chain. Indeed, *IDH*-mutant CCAs were shown to exhibit high levels of mitochondrial and low levels of chromatin modifier gene expression, such as low *ARID1A* expression due to DNA hypermethylation^121^. Besides epigenetic silencing, inactivating mutations in multiple chromatin-remodelling genes (including *BAP1*, *ARID1A* and *PBRM1*) are common in iCCA^151^.

Developmental pathways, including Notch, WNT and transforming growth factor-β (TGFβ) signalling pathways are prominently active in iCCA compared with HCC, as shown by integrated microarray analysis^152^. During liver repair and in inflammatory conditions (known risk factors for iCCA), signalling pathways involved in biliary development are activated in ductular reactive cells, including Notch, WNT, Hippo–YAP and Hedgehog. The Notch pathway is known to be involved in biliary repair, growth, tubulogenesis, fibrosis and maintenance of the stem cell niche; defective Notch function due to *JAG1* or *NOTCH2* mutations causes impaired regeneration and Alagille syndrome^153^, whereas increased Notch activity has been associated with primary liver tumours^154^. Overexpression or aberrant Notch receptor expression has been reported both in iCCAs and eCCA, including pCCA and dCCA^155–157^. Activation of Notch signalling was shown to mediate transdifferentiation of hepatocytes into cholangiocytes during carcinogenesis^79–81,158^. In this regard, experimental overexpression of the intracellular domain of NOTCH1 receptor (NICD1) in hepatocytes has been associated with the development of iCCA in mouse models^79,80,158^. Similarly, inhibition of NOTCH2, the expression of which has been shown to be related to well-differentiated iCCA^155^, markedly reduced tumour burden in various mouse models of liver cancer (including iCCA)^81,159^, whereas overexpression of NOTCH3 was associated with the development and progression of iCCA, promoting cell survival via PI3K–AKT signalling^160^. Several Notch inhibitors are being developed, and their availability increases interest in this pathway^161^.

The WNT–β-catenin signalling pathway is also known to be activated in most CCAs, in part as an effect of the release of Wnt ligands by inflammatory macrophages infiltrating the stroma^162,163^, but also as a consequence of DNA methylation alterations targeting this pathway^133^ and/or mutations encoding key components of the canonical WNT–β-catenin signalling pathway^164^. Notably, the promoter of the WNT–β-catenin pathway inhibitor SOX17 was hypermethylated in CCA tumour tissue compared with healthy tissue, correlating with a worse prognosis after tumour resection^132^. Noteworthy, SOX17 was shown to regulate cholangiocyte differentiation and to act as a tumour suppressor in CCA in vitro^132^. WNT inhibitors successfully inhibit tumour growth in experimental models^163^ and clinical trials with agents targeting this pathway are currently being explored^164^. The Hippo–YAP signalling pathway regulates organ size and cell proliferation, among other functions^165^. YAP is a transcriptional co-activator that is usually inhibited by Hippo (MST1 or MST2), but can be activated by Hippo-independent signals, such as inflammation and changes in ECM composition and stiffness^166^. Several groups have reported increased nuclear expression of YAP in CCA specimens and correlation with a worse prognosis^167–169^. In vitro studies on CCA cell lines have shown that YAP can be activated by IL-6, PDGF and fibroblast growth factor^170,171^. PDGF and fibroblast growth factor form a feed-forward loop activating YAP; YAP transcriptional targets are genes of these signalling pathways, such as *FGFR1*, *FGFR2* and *FGFR4* (refs^170–172^). Genetic alteration of the YAP pathway seems to be uncommon in CCA, according to an integrative genomic analysis of CCA specimens^121^. However, mutations in *ARID1A* have been reported in up to 14% of CCAs^149^. *ARID1A* encodes a subunit of the SWI–SNF chromatin-remodelling complex that among other functions reduces YAP transcriptional activity^173^.

## EMT, stemness and plasticity

EMT is a cell plasticity-promoting phenomenon initially reported to occur during embryogenesis, but that also takes place in cancer, enabling epithelial cancer cells to acquire mesenchymal features with invasive properties that lead to metastatic colonization^174^. The prototype inducer of EMT is the TGFβ-dependent pathway, whose signature has been identified in iCCA stroma^8,175^. In CCA, TGFβ induces EMT directly or cooperates with other major EMT inducer pathways such as EGFR^176,177^. During this plastic EMT programme, tumour cells lose their epithelial traits and gain mesenchymal features^178^. Although initially considered as a binary process, it is now well established that epithelial cells undergoing EMT become mesenchymal in a gradual manner, known as partial EMT^178,179^. Thus, EMT is a dynamic process that gives rise to intermediate cellular states with both epithelial and mesenchymal traits, contributing to cell heterogeneity and a broad range of functions from cancer initiation to progression^178,179^. Notably, EMT is orchestrated by transcription factors (EMT-TFs), comprising SNAIL, ZEB and TWIST family, that regulate the expression of epithelial and mesenchymal genes^180^. CCAs express EMT-TFs, which are associated with poor prognosis regardless of anatomical localization^181^. Beyond the EMT programme, EMT-TFs display pleiotropic roles linking EMT to stemness, metabolic reprogramming, immune evasion and drug resistance^178,182,183^.

Increasing evidence suggests associations between EMT and acquisition of cancer stem cell (CSC) properties in different cancer types^65,184^, and this might also contribute to CCA heterogeneity as well as resistance to anticancer drugs. Importantly, CSCs represent a peculiar subcompartment of the tumour cell population crucially involved in recurrence, metastasis and drug resistance^185–187^. A growing body of evidence indicates that CSCs express EMT traits in human CCAs^65,187–189^. Interestingly, CCA emerging in patients with PSC are characterized by EMT features and high expression of stem and/or progenitor cell markers in peribiliary glands, suggesting a connection between EMT and stemness in tumour initiation^49^. Indeed, EMT-TFs, such as ZEB1, regulate expression of CSC markers by inhibiting miR-200 family members, well-known potent stemness repressors^190^. In stem-like iCCA, a signature linking miR-200c with EMT regulators such as ZEB1 and TGFβ has been identified^191^. Besides EMT, TGFβ is known to promote stemness in CCA cells in vitro (human CCA cell line TFK-1)^192^. A statistically significant correlation between TGFβ1 and aldehyde dehydrogenase 1 (ALDH1), a functional CSC marker, has been found in both iCCA and eCCA^192^. Furthermore, TGFβ-induced EMT resulted in acquisition of mesenchymal traits, ALDH expression and resistance to 5-fluorouracil (5-FU) in vitro^192^. Moreover, new evidence suggests that cell plasticity promoted by the EMT programme confers immunosuppressive effects on carcinoma cells by mechanisms not completely understood^178^; one mechanism identified so far is the regulation of the immune checkpoint PD1 ligand (PDL1) by ZEB1 in breast cancer cells^193^.

## Tumour microenvironment

**Fig. 6:**
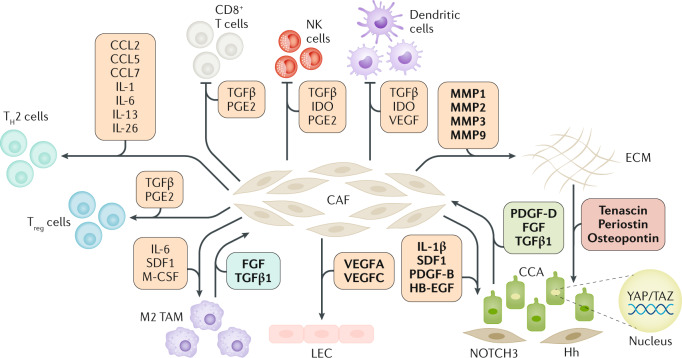
Central role of cancer-associated fibroblasts in promoting tumour growth and metastasis of cholangiocarcinoma. Cancer-associated fibroblasts (CAFs) are recruited and persistently activated by cholangiocarcinoma (CCA) cells, in response to the effects of PDGF-D, and of FGF and TGFβ1, also released by tumour-associated macrophages (TAMs). In turn, CAFs enhance cell proliferation and the invasive ability of CCA cells directly, or by influencing the activity of other cells in the tumour microenvironment. CAFs stimulate tumour-associated lymphangiogenesis (lymphatic endothelial cell (LEC)), support M2 polarization of TAMs and the activation of regulatory T (T_reg_) cells, while dampening the activity of CD8^+^ T cells, natural killer (NK) and dendritic cells. CAFs also induce heavy remodelling of the extracellular matrix (ECM), which becomes stiffer and affects mechanotransduction of CCA cells, leading to activation of intracellular pathways, including YAP–TAZ. Soluble factors mediating each cell–cell interplay are shown in boxes of different colours according to their origin (orange from CAFs, green from CCA cells, light blue from TAMs, red from ECM). Mediators in bold are those with proven effects, the rest are putative signalling molecules. CAF-derived short-range (Hedgehog (Hh)) and direct (NOTCH3) cell–cell developmental cues also underlie interactions with CCA cells (lower right corner).T_H_2 cell, T helper 2 cell.

CCA tumours contain a diverse range of cellular types (Fig. 6). Although the tumour epithelium is considered as the coordinator of tumour growth, the importance of the TME cannot be understated. Histopathologically, CCA is typified by an extensive cellular and acellular stroma that can comprise the bulk of the tumour^194^. CCA shares many characteristics with scars that form around bile ducts in premalignant disease, as usually found in PSC and congenital hepatic fibrosis, suggesting that the origin of the tumour stroma can be found in the regenerative microenvironment during bile duct repair^195^. The CCA stroma consists of cancer-associated endothelial cells, CAFs and a complex group of inflammatory cells, including macrophages, neutrophils, natural killer (NK) and T cells^196^. In addition to this complex cellular microenvironment, the tumour stroma also contains an extensive network of ECM proteins such as collagens, laminin and fibronectin^197,198^. The TME directly interacts with the cancer epithelium to support epithelial proliferation and tumour growth, among which CAFs have been the most extensively investigated.

### Cancer-associated fibroblasts

CAFs are a heterogeneous population of spindle-shaped cells with mesenchymal origin that contribute to tumour progression in many human cancers^199^. In CCA, the abundance of CAFs positively correlates with tumour growth and poor survival^200^. CAFs most likely originate from several different cells types, namely tissue-resident portal fibroblasts, hepatic stellate cells, pericytes, bone marrow-derived mesenchymal stem cells and monocyte precursor-derived fibrocytes via transdifferentiation and activation^181,201,202^. This activation process also results in a metabolic reprogramming that enhances proliferation, cellular motility, as well as secretion of regulatory molecules and components of the ECM. Importantly, although CCA cells express mesenchymal markers they do not transdifferentiate into CAFs, but they do secrete PDGF-D to stimulate fibroblast migration^203^. In CCA, the persistent activation of fibroblasts is induced primarily by TGFβ, fibroblast growth factor and PDGF, which are released from tumour-associated macrophages and CCA cells^204^. TGFβ was reported to be pivotal in promoting an iCCA-desmoplastic phenotype in a 3D rat organotypic culture model^205^, and targeting the TGFβ pathway in thioacetamide-treated rats improved fibrosis and reduced CCA burden^206^. CAFs secrete a multitude of signalling molecules (such as IL-1β, PDGF-B, heparin-binding EGF-like growth factor and SDF1) that promote cancer progression by enhancing proliferation, survival, chemotaxis and angiogenesis^148^. Furthermore, CAFs have also been shown to promote CCA growth through short-range and direct cell–cell morphogenetic signals, such as NOTCH3 (ref.^160^) and Hedgehog^207^. By secreting immunomodulatory factors, CAFs can also promote an immunosuppressive TME^208^: they regulate innate immunity by supporting M2 macrophages, and decreasing NK cell activation. Regarding adaptive immunity, CAFs promote regulatory T cells and T helper 2 cells, and disable dendritic cells and cytotoxic T cells^208^. Data support the ability of CAFs to interact with lymphatic endothelial cells^209^. Following stimulation by PDGF-D originated from the tumoural cholangiocytes in vitro, CAFs secrete VEGF-A and VEGF-C, which recruit and assemble lymphatic endothelial cells in vascular structures susceptible to tumour cell intravasation^209^.

Cell interactions within the TME are favoured by the ECM, which is gradually ‘transformed’ into a compact and stiff scaffold, enabling mutual communications and exchange of paracrine factors between the different cell elements^210^. The ECM is continuously remodelled by deposition of newly synthesized matricellular proteins, including tenascin C, osteopontin and periostin, in concert with an intensive degradation by matrix metalloproteinases (MMPs; MMP1, MMP2, MMP3 and MMP9) that are copiously released by CAFs, tumour-associated macrophages and malignant cholangiocytes^210^. Thanks to these phenotypic changes, ECM boosts key pro-invasive functions of tumour cells. In cooperation with collagen I, tenascin C and integrins (α5β1, α5β3, α5β5 and α6β4), periostin stimulates cell proliferation of malignant cholangiocytes in a PI3K–AKT-dependent manner in vitro^211^. Increased ECM stiffening is also instrumental in the activation of intracellular mechanosensors, such as YAP–TAZ, involved in tumour initiation and progression. Whereas soft ECM inhibits YAP–TAZ activity by favouring its sequestration by the SWI–SNF chromatin-remodelling complex through ARID1A, stiff ECM induces YAP–TAZ to detach from SWI–SNF and to bind to TEAD, unfolding a transcriptional programme and promoting cell proliferation, CSC traits, plasticity and reprogramming^173,212^. Overall, the multifaceted interplay of CAFs with tumour cells, immune cells, lymphatic endothelial cells and ECM is continuously evolving (Fig. 6) and could offer potential therapeutic targets. Importantly, selective pro-apoptotic targeting of CAFs with subsequent reduction in tumour growth and lymph node metastases has been demonstrated in a CCA rat model^213^. Overall, the signalling networks that govern CCA tumours are the result of the intrinsic genomic and epigenetic alterations of tumour cholangiocytes, as well as their interplay with CAFs, immune cells and ECM. The secretion of proinflammatory, oncogenic and fibrogenic factors from CCA cells could contribute to the recruitment of other cells to the TME, which in turn will activate and sustain specific signalling pathways in cancer cells, thus perpetuating CCA growth and progression.

### Immunobiology

Transcriptomic sequencing of CCA tumours has demonstrated that the subset of patients with the poorest prognosis have an elevated tumour mutational load and enhanced expression of immune checkpoint molecules^56^. Importantly, the presence of T cell-infiltrated TMEs, characterized by infiltration of CD8^+^ T cells, chemokines and molecules responsible for T cell priming and immune infiltration, is associated with higher response to immune checkpoint blockade, whereas non-T cell-infiltrated TMEs have poorer responses^214,215^.

Regarding innate immune responses, activated or ‘M2-like’ tumour-associated macrophages are anti-inflammatory and immunosuppressive. M2-like macrophages stimulate WNT signalling with consequent CCA progression^163^, and are associated with inferior patient outcomes^216,217^. High numbers of M2 tumour-associated macrophages are linked to poor disease-free survival in patients with iCCA^217^. Similarly, in a retrospective study of patients with pCCA who had undergone surgical resection, high density of tumour-associated macrophages in the tumour invasive front correlated with increased local and tumour recurrence^216^. Myeloid-derived suppressor cells are another immunosuppressive element in the TME. Fibroblast activation protein-positive (FAP^+^) CAFs promote myeloid-derived suppressor cell infiltration in desmoplastic tumours^218^. Moreover, increased stromal FAP expression in human resected CCA specimens has been linked to poor patient outcomes^218^. The presence of CD83^+^ dendritic cells in human resected CCA specimens was associated with better outcomes^219^. Although NK cells comprise 30–40% of all hepatic lymphocytes^220^, current knowledge on the role of these cells in CCA is limited. Culture of CCA cells (human CCA cell lines, Hucct1 cells and OZ cells) with the anti-EGFR monoclonal antibody cetuximab augmented CCA cell death via NK cell-induced antibody-dependent cellular cytotoxicity^221^. Similarly, infusion of ex vivo-expanded human NK cells in CCA mouse xenograft models resulted in tumour regression^222^.

CCA progression has been associated with a decrease in the components of the adaptive immune response^223^. Immunohistochemical analyses have demonstrated a preponderance of CD8^+^ T cells within the tumour and CD4^+^ T cells in the tumour–liver interface^224^, as well as an association with longer OS and the presence of tumour-infiltrating CD4^+^ or CD8^+^ T cells^223,225–227^. Similarly, the presence of B cells has been linked to a favourable prognosis in CCA^223,224^. Factors associated with a higher likelihood of response to immune checkpoint blockade include the presence of biomarkers such as PDL1, genetic aberrations such as DNA mismatch repair (MMR) deficiency and/or microsatellite instability (MSI), and the cumulative tumour mutational burden^2^. On the basis of small cohorts of patients with CCA (range 41–104 patients across the studies), PDL1 is expressed in 42–72% of tumours^228–230^, and seems to be present primarily on immune cells^228,229^. MMR deficiency has been reported in 5% of pCCA or dCCAs and 10% of iCCAs^231^. Of note, hypermutation was found in 6% of CCAs and MMR deficiency and/or MSI was present in 36% of these hypermutated tumours^56^. MSI-high tumours are generally ‘hot’ tumours with an increased number of neoepitopes, CD8^+^ T cell infiltration, and improved responses to immune checkpoint blockade in cancer generally^215^. In a cohort of 86 patients with MMR-deficient tumours, including four patients with CCA, immune checkpoint blockade with the anti-PDL1 monoclonal antibody pembrolizumab resulted in a complete response in one of the patients with CCA and stabilization of disease in the other three^232^. These data indicate that immune-directed therapies including immune checkpoint blockade are a promising approach for, at least, this subset of patients with CCA.

## In vitro and in vivo experimental models

Over the past decade, a number of in vitro and in vivo models of cholangiocarcinogenesis have been generated to clarify the phenotypic, biochemical and biological events occurring during the transformation of normal cells into fully malignant cholangiocytes (Table 3). In vitro cell lines, mainly derived from human CCA specimens, have been used widely as a tool to study this disease^76,233–235^. Cell lines exhibit various advantages over animal models: they are free from non-tumourous and necrotic tissues, their growth can be synchronized, relatively high numbers of cells can be produced, cell proliferation and apoptosis can be accurately determined, and they can be molecularly modified (that is, by overexpression or silencing of genes, using antisense oligonucleotides, small interfering RNAs, CRISPR–Cas, and so on), therefore enabling the study of single genes or signal transduction pathways. Furthermore, cell lines can be subjected to drug administration. However, in vitro passaging renders cell lines increasingly different from the original tumours. Primary cultures of CCA cells from tumour tissue are used shortly after derivation and grown under serum-free growth factor-enhanced conditions; therefore, more closely resembling the in vivo situation^236,237^. Unfortunately, important shortcomings also apply to this system; in particular, primary cultures are time-consuming and elimination of non-tumour cells can be complicated. Furthermore, primary cultures can only be established from surgically resected specimens, limiting the applicability to a subset of patients with CCA who have undergone surgery. Also, primary culture cells lack realistic intercellular and cell–matrix interactions^236,237^. Importantly, preneoplastic (for example, PSC-derived cholangiocytes) and/or normal cholangiocyte primary cultures should be used as controls^132,140,238,239^.

**Table 3:** In vitro and in vivo models of cholangiocarcinoma

Models	Main features	Advantages	Limitations	Examples	Refs
***In vitro models***					
Cell lines	A permanently established cell culture that proliferates indefinitely given appropriate fresh medium and space	Devoid of non-neoplastic and necrotic tissues; growth can be synchronized; high number of cells generated; easy assessment of proliferation and cell death; possibility of genetic manipulation (overexpression, silencing) and drug administration	Become different from original tumours following in vitro passages; generally representing only advanced tumour; lack of TME (immune cells, stromal cells and blood vessels); genetically unstable; normal cholangiocyte cultures should be used as control	Human (HuCC-T1 KKU-156, Mz-ChA-1, TFK-1, QBC939, etc.); mouse (SB1-SB7); rat (CGCCA) CCA cell lines	^76,233–235,405,406^
Primary cultures	Cell culture system that is formed by culture cells directly obtained from CCA tissues	More similar than cell lines to the in vivo situation	Labour-intensive; only generated from surgically resected specimens; lack of realistic cell–cell and cell–matrix interactions	Primary cultures obtained from human or rodent (mice and rats) resected CCA specimens	^236,237^
Spheroids	Cell aggregates that are either grown in suspension or embedded in a 3D matrix using 3D culture methods	Mimic spatial architecture, physiological responses, secretion of soluble mediators, gene expression patterns and drug resistance mechanisms of CCA	Long-term culture difficult	Human CCA spheroids in 3D culture; 3D rat CAF–CCA cell co-culture models	^205,240,241,407^
Organoids	Simplified and ‘miniaturized’ version of an organ generated in vitro in 3D and preserving the tissue of origin	Accurately mimic genetics, cell organization and behaviour, and response to drugs or mutations, in a setting that resembles the original microenvironment; allow the study of the various phases of carcinogenesis; can be grown from a limited amount of starting material (biopsy samples); useful for gene editing	Lack of circulation limits their size and complexity; accuracy of the various phases of cancer development still need to be fully validated in these 3D structures	Organoids of CCA isolated from human or rodent (mice and rats) liver specimens	^76,242,243^
***In vivo models***					
Chemically- and infestation-induced models	Mice, rats or Syrian hamsters subjected to the administration of chemical carcinogens via various sites and modalities	Enable the identification of natural or occupational carcinogens; tumour onset and progression easy to assess from early stages; presence of chronic inflammation; ‘natural’ microenvironment and intact immune system	Different pharmacokinetics and drug metabolism from humans; potential drug toxicity; difficult to identify the driving pathogenetic events; development of cholangiofibrosis and intestinal metaplasia preceding CCA occurrence in TAA and Furan models; monitoring of carcinogenesis using the same instrumentation as in humans (CT scan, MRI)	TAA Furan *Tp53*ko–CCl4; diethylnitrosamine; dimethylnitrosamine; *Opisthorchis viverrini*	^248,408^
Genetically-engineered mouse models (GEMM)	Mice whose genome has been altered using genetic engineering techniques	Tumour onset and progression easy to assess from early stages; possible to engineer specific mutations to study gene function or to add reporters; well-established technology; amenable to genetic screening approaches; tumours develop in the presence of an intact immune system and a proper tumour microenvironment; able to predict the response of human tumours to therapy	Mouse strains do not represent the genetic diversity of the human population; mouse tumours grow very fast relative to human tumours; the engineering strategies are complicated and expensive, requiring a dedicated infrastructure; lack of chronic inflammation in the background; monitoring of carcinogenesis using the same instrumentation as in humans (CT scan, MRI)	Alb‐Cre;*Smad4*f/f;*Pten*f/f Alb‐Cre;*Kras*^LSLG12D/+^;*Pten*f/f Alb‐Cre;*Kras*^LSLG12D/+^;*Tp53*f/f Alb‐Cre;*Kras*^LSL‐G12D/+^;*Fbxw7*^LSL‐ R468C^ Alb‐Cre;*Idh2*^LSL‐R172^;*Kras*^LSL‐ G12D^ Alb‐Cre;*NotchIC* Alb‐Cre;*Tp53*f/f;*NotchICD*	^248,408^
Implantation models	Mice or rats in which the tumour component from an external source (cell lines, human tissues, etc.) is implanted either in the analogous (orthotopic) or a different (ectopic) organ from the original	Easy to generate and inexpensive; recapitulate some of the human tumour features	Useful mainly for the study of advanced tumour stages; mainly stable at the genetic level; different tumour microenvironment from the native condition and lack of immune cells	Subcutaneous xenografts of human (Mz-ChA-1, QBC939, etc.) or mouse (SB1-SB7) cell lines in nude or syngeneic mice; patient-derived xenografts in female NOD/SCID mice; bile duct inoculation of tumorigenic rat cholangiocyte cell lines	^205,248,406,409^
Transposon-based models	Mice in which a gene or a combination of genes is stably integrated into the hepatocytes integrated using a transposase	Tumour onset and progression easy to assess from early stages; possible to deliver specific mutations to study gene function or to add reporters; easy, inexpensive, fast, and high-reproducible technology; amenable to genetic screening approaches; tumours develop in the presence of an intact immune system and a proper tumour microenvironment; allow prediction of the response of human tumours to therapy	Mouse tumours grow very fast relative to human tumours; CCA develop from mature hepatocytes and not from cholangiocytes or progenitor or stem cells; monitoring of carcinogenesis using the same instrumentation as in humans (CT scan, MRI)	*NRAS*^V12^;*Ink4A*;*Arf*^−/−^ *PIK3CA*;*Yap* *NICD1* *NICD1*;myr*AKT* *YAP*^S127A^;myr*AKT* *NRAS*^V12^;myr*AKT* *NICD1*;*KRAS*^LSLG12D+^ *JAG1*;myr*AKT* *YAP*^S127A^;myr*AKT* + IL-33 injection	^247,248,408^

CAF, cancer-associated fibroblast; CCA, cholangiocarcinoma; TAA, thioacetamide; TME, tumour microenvironment.

To recapitulate more adequately the in vivo tumour tissue structure and to investigate the interaction between CCA and the TME, 3D model systems, known as tumour spheroids and organoids, were developed^240,241^. Tumour spheroids are self-assembled cultures of cancer cells in the presence or absence of stromal cells within a hydrogel, mimicking the basement membrane, where cell–cell interactions predominate over cell–substrate interactions^241^. By contrast, tumour organoids are self-organizing stem cell-like structures cultured and expanded in a hydrogel^76,242,243^. Organoids are successfully established from resected tissue biopsy and needle biopsy samples, faithfully recapitulating the patient tumour at the histopathological level, both in culture and as xenografts in immune-deficient mice^242,244^. However, to recapitulate CCA tumours in vivo, organoids should be co-cultured with stromal cells. Importantly, whole-exome sequencing revealed that the vast majority of the mutations are retained in liver cancer organoids derived from resected tissues, whereas mutation retention is heterogeneous in biopsy-derived liver cancer organoids^76,242,243^. Furthermore, CCA organoids have been shown to be a reliable system for drug testing and personalized medicine applications, and possess an almost negligible capacity to differentiate into hepatocytes^76,245^. As an alternative method, CCA organoids can be established by inducing genetic mutations in healthy organoids via viral transduction and CRISPR–Cas9 genome editing approaches, thus enabling the characterization and elucidation of the roles of oncogenes and/or tumour suppressor genes, either alone or in combination, in cholangiocarcinogenesis^76,246^.

Mouse models of CCA enable the investigation of the pathobiology of the disease and treatment response in a context that more closely recapitulates the human disease^76,247–249^. Multiple approaches have been used to induce CCA formation in mice and the principal mouse models can be classified into four major groups: chemically induced models, in which a chemotoxic drug is responsible for the oncogenic insult(s); genetically engineered mouse models (GEMM); implantation models; and transposon-based models. As human CCA can develop in the setting of a diseased liver, various methods have been developed to mimic liver alterations, such as those induced in humans by viral hepatitis, chronic inflammation and cholestasis, further increasing the similarity with the human situation. Another major advantage of in vivo models is that they enable the study of CCA starting from early pre-neoplastic to fully progressed lesions, meaning researchers can dissect the specific molecular events occurring at various stages of cholangiocarcinogenesis. In addition, in vivo models enable real-time monitoring of tumour development and response to therapies using imaging modalities such as CT or MRI, or other techniques involving bioluminescence.

## Diagnostic and prognostic biomarkers

The current ‘omics’ era is enabling the discovery of new and promising biomarkers in biofluids (serum, urine, bile, saliva) and tumour tissue that could change the paradigm in disease diagnosis and management in the upcoming years (Supplementary Figure 1).

Circulating nucleic acids found in biofluids after active transport or resulting from dying cells are promising diagnostic and prognostic tools for human disorders^250–252^. Cell-free DNA (cfDNA) has been envisaged as mirroring changes in tumour aggressiveness and size, being found both in tumour tissue and plasma from patients with CCA^253^. Detection of cfDNA in plasma samples could also guide potential mutational-based therapeutic interventions as de novo multiple point mutations in *FGFR2* kinase domain were detected in cfDNA, primary tumours and metastases from patients with CCA with acquired resistance to the pan-FGFR inhibitor BGJ398 (ref.^254^). On the other hand, miRNAs have received special attention due to their increased stability and abundance in biofluids. Two meta-analyses have evaluated their diagnostic value for CCA, and found a pooled area under the receiver operator curve (AUC) of ~0.9 (refs^255,256^). Notably, bile represented the biological fluid with the highest diagnostic capacity, followed by serum, tissue and urine (AUC 0.95, 0.913, 0.846 and 0.745, respectively)^256^. In this regard, some bile miRNAs have already been shown to display increased diagnostic capacity for CCA, in comparison with healthy individuals (miR-9, miR-145)^257^ and also when comparing patients with PSC-derived CCA and isolated PSC (miR-412, miR-640, miR-1537, miR-3189)^258^. Importantly, combining miR-1537 with CA19-9 resulted in higher diagnostic values than CA19-9 alone (AUC 0.91 versus 0.88; *P* > 0.05)^258^. In serum, the levels of miR-21 (refs^259–261^), a well-known onco-miR, were found to be increased in patients with CCA, compared with healthy individuals, positively correlating with clinical stage and poor survival, although the translation of this miRNA into clinics should be performed carefully since it is usually increased in serum and/or plasma of patients with HCC and other liver diseases and cancers^262,263^. Other miRNAs were also differentially found in the serum and/or plasma of patients with CCA compared with control individuals^264–270^, but with some inconsistency, which underscores the necessity for conducting further studies for validation in large, biopsy-proven and well-characterized cohorts of patients and adequate controls.

Proteins and cytokines are now regarded as potential diagnostic and/or prognostic biomarkers. A soluble fragment of cytokeratin-19 (CYFRA 21-1), MMP-7, osteopontin, periostin and IL-6, among others, were shown to be enriched in the serum of patients with CCA, when compared with healthy individuals as controls and/or patients with benign biliary diseases (such as PSC)^271–282^. Among these biomarkers, increased CYFRA 21-1 and osteopontin levels showed superior diagnostic capacity for identifying CCA compared with CA19-9 and CEA^271,275^, and also showed prognostic value. Of note, increased serum periostin levels were also associated with decreased OS, and the serum periostin level was an independent prognostic factor (HR 3.197)^282^. As cancer cells display marked metabolic alterations, measuring metabolites in distinct biological samples is now regarded as an encouraging alternative to find diagnostic and/or prognostic biomarkers. Up to now, only a limited number of studies have addressed this issue. Bile acids and phospholipids have been highlighted as promising metabolites in bile for the diagnosis of CCA, as their levels are increased in patients with CCA compared with healthy individuals and patients with HCC^283–287^. Serum metabolomics has also revealed promising diagnostic biomarkers^288,289^. An international collaborative study including patients with biopsy-proven iCCA, HCC or PSC and healthy individuals found that several metabolites had higher diagnostic capacity for iCCA than CA19-9, and the authors proposed an algorithm containing six metabolites that was able to differentially diagnose iCCA and HCC (AUC 0.9) in discovery (*n* = 20 per group) and validation phases (independent cohorts of 14–15 patients per group)^289^. Interestingly, proteomic analysis of serum extracellular vesicles from patients with CCA, HCC or PSC and healthy individuals as controls revealed candidate proteins with high accuracy for the differential diagnosis of these liver diseases, having higher AUC values than either CA19-9 or α-fetoprotein levels^239^. Furthermore, another study identified an extracellular vesicle-derived miRNA panel in bile (miRNAs miR-191, miR-486-3p, miR-1274b and miR-484) for the discrimination of CCA from non-malignant biliary diseases^290^. Although few studies have addressed the potential role of circulating tumour cells (CTCs) as diagnostic and/or prognostic biomarkers in CCA, in a study investigating the associations between numbers of CTCs, patient and tumour characteristics and survival in patients with biliary tract cancer, 17–25% of the patients showed elevated numbers of CTCs (two or more per 7.5 mL of blood)^291–293^, and <10% of the patients showed five or more CTCs per 7.5 mL of blood^292^. Elevated numbers of CTCs were correlated with greater tumour extent and with reduced overall and disease-free survival^292,293^. Nevertheless, novel isolation techniques are warranted since the available ones are mainly based on the presence of epithelial cell adhesion molecule, which is elevated in only ~10–20% of CCAs^291^.

Specific biomarkers in tumour tissue represent promising tools to predict prognosis and treatment response to potential adjuvant therapies in resected CCAs. In two large and independent cohorts of patients with iCCA who had undergone tumour resection (*n* = 137 in one study^56^; *n* = 292 in the other^119^), mutations in *KRAS* (12–16%) and *TP53* (13–20%) were associated with shorter OS and an increased rate of tumour recurrence when compared with patients with *IDH1* or *IDH2* mutations or an ‘undetermined’ group (with none of the aforementioned mutations)^56,119^. According to the transcriptomic profile of iCCA tumours^110^, the proliferation type was linked with a worse prognosis. Furthermore, a specific 36-gene signature was strongly associated with poor survival in patients with resected iCCA^8^, and a meta-analysis of 73 studies (including 4,126 patients with CCA) revealed 77 prognostic protein biomarkers, of which fascin, EGFR, mucin 1 (MUC1), MUC4 and p27 were independently associated with OS: high levels of EGFR, MUC1, MUC4 and fascin expression were associated with reduced survival, whereas a high level of p27 expression was associated with increased survival^294^. Increased levels of miR-21 expression in iCCA were also positively correlated with clinical stage at diagnosis, tumour differentiation status and were linked with poor overall and progression-free survival^259,295^.

## Management

### Treatment of localized and advanced disease stages

**Fig. 7:**
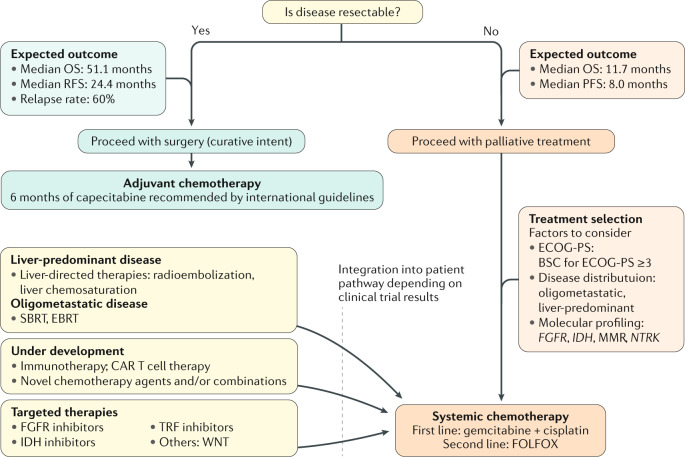
Current decisions and management of patients with cholangiocarcinoma. Flow chart of the presentation, management and outcome of patients with cholangiocarcinoma (CCA) according to current formal guidelines (Supplementary Table 1). BSC, best supportive care; CAR, chimeric antigen receptor; EBRT, external beam radiation therapy; ECOG-PS, Eastern Cooperative Oncology Group Performance Status; FOLFOX, folinic acid, 5-fluorouracil and oxaliplatin; MMR, DNA mismatch repair; OS, overall survival; PFS, progression-free survival; RFS, relapse-free survival; SBRT, stereotactic body radiation therapy.

Surgery is a potential curative option for CCA. However, most patients (∼70%) are diagnosed at late stages due to lack of specific symptoms^104^. When disease is unresectable, only palliative treatment is possible^104,296^. Figure 7 summarizes the experience and recommended management of patients diagnosed with CCA according to current guidelines, and lists upcoming potential treatments (see also Supplementary Table 1 for a summary of relevant clinical trials of drugs for CCA).

#### Surgery

Most patients with CCA have metastatic or locally advanced (that is, unresectable) disease at presentation, and only ∼25% are eligible for resection^297^.

The majority of patients with iCCA present with large tumours (median size 6 cm)^298^. In ∼15% of patients the tumour grows towards the hepatic hilum causing biliary obstruction. A biopsy is not needed to confirm the diagnosis in patients with characteristic CCA imaging, elevated serum levels of CA19-9 and normal IgG4 levels, or after excluding other primary tumours (that is, colorectal, gastric and breast). The goal of surgery is a complete margin-negative resection (R0) with an adequate future liver remnant. Most patients require an (extended) hemi-hepatectomy with lymphadenectomy of at least six locoregional lymph nodes for adequate staging^103^. Staging laparoscopy is recommended by clinical guidelines, especially in patients with a high CA19-9 level or major vascular invasion^299^.

Regarding pCCA, pre-operative drainage of the future liver remnant is performed to improve liver function and avoid post-hepatectomy liver failure^300,301^. For this purpose, imaging (CT and/or MRCP) should be performed prior to biliary drainage for accurate staging and surgical planning. Surgery typically involves an (extended) hemi-hepatectomy, including the caudate lobe with en-bloc resection of the extrahepatic bile duct and regional lymph nodes. Staging laparoscopy should precede laparotomy to exclude occult metastatic disease that occurs in ∼15% of patients^302^. The 90-day postoperative mortality is up to 10% in experienced centres in Europe, with most (~48%) of those who die dying from post-hepatectomy liver failure^303,304^. In the largest centre in Asia, overall mortality was 4.7% for the period 1977–2010, with the rate markedly decreasing from 11.1% to 1.4% for the periods 1977–1990 and 2006–2010, respectively^305^. Patients with metastatic pCCA clearly do not benefit from resection^305^. However, patients with locally advanced disease undergo resection. The presence of Bismuth type IV pCCA (involving both the right and left intrahepatic ducts) is no longer an absolute contraindication for complete resection since it is associated with an OS similar to that in patients with less extensive biliary extension^306^. Moreover, resection and reconstruction of the portal vein and hepatic artery are increasingly performed^307,308^. However, tumour abutment on imaging of the main portal vein or common hepatic artery exceeding 180° is associated with a poor prognosis^297^. In most patients with extensive vascular involvement, the small potential benefit of resection might not justify the considerable surgical mortality rate. Future research should improve pre-operative assessment of the biliary extent of pCCA to reduce the number of R1 resections and of the function of the future liver remnant to reduce the likelihood of post-hepatectomy liver failure. Surgical strategies for dCCA usually require performing a pancreaticoduodenectomy, with removal of the head of the pancreas, the first part of the duodenum, the gallbladder and the bile duct^296^.

Patients with distant metastatic disease or involvement of aortocaval or truncal nodes are unlikely to benefit from resection^309^. In a 2018 SEER analysis, even patients with positive regional lymph nodes had similar OS after resection and adjuvant systemic chemotherapy^310^. Most guidelines recommend resection only for solitary tumours^28,296,299^. In a study investigating long-term outcomes after resection of iCCA, the median OS in patients with a solitary iCCA was 43.2 months, versus 21.2 months in those with two tumours and 15.3 months in those with three or more tumours^311^. Patients requiring major vascular resection for iCCA increasingly undergo resection with an acceptable median OS of 33 months^312^.

#### Resectable disease: role of adjuvant therapy

Frequent post-surgical relapse^313,314^ has led to multiple attempts to identify patients at increased risk of relapse^315,316^ and also to a number of studies of adjuvant therapy. Three phase III randomized clinical studies have been reported, and in all of them patients with resected biliary tract cancer (CCA and gallbladder cancer) were randomly assigned to observation alone or chemotherapy^317–319^. The chemotherapy arm was gemcitabine in the BCAT study (pCCA or dCCA only)^317^, gemcitabine and oxaliplatin (all biliary tract cancers) in the PRODIGE-12 study^318^, and capecitabine in the BILCAP study (all biliary tract cancers)^319^. A total of 226, 196 and 447 patients were randomly assigned in each study, respectively^320^. Although the BCAT and the PRODIGE-12 study failed to show a benefit from gemcitabine-based chemotherapy, the BILCAP study showed a benefit from adjuvant capecitabine in the pre-planned sensitivity analysis when compared with observation alone, in terms of OS (HR 0.71); however, no statistically significant benefit was observed in the intention-to-treat OS analysis. The BILCAP study did show a benefit in favour of capecitabine in terms of relapse-free survival (HR 0.75). Based on the partial benefits reported in the BILCAP trial, international guidelines published in 2019 recommend adjuvant capecitabine for a period of 6 months following curative resection of CCA as the current standard of care^321^. The role of chemoradiotherapy remains unclear and might be of benefit in patients with pCCA or dCCA with microscopic positive surgical margins (R1)^321,322^ or other high-risk factors, although this approach needs to be confirmed in prospective studies. Ongoing studies are evaluating the role of combination chemotherapy such as cisplatin and gemcitabine (ACTICCA-1 trial, NCT02170090; ClinicalTrials.gov) in the adjuvant setting.

#### Liver transplantation for intrahepatic and perihilar CCA

The inability to obtain a complete resection remains a limitation. Liver transplantation for pCCA was initially determined to be contraindicated due to a high rate of recurrence (~50%)^323–325^. However, following promising initial single-centre reports, a multicentre retrospective study in 216 patients with early-stage, unresectable pCCA treated with neoadjuvant chemoradiotherapy followed by liver transplantation in 12 centres in the USA demonstrated 5-year disease-free survival of 65%, with an intent-to-treat 5-year survival of 53%^326–328^. An area of uncertainty is that in a subgroup of patients, no malignancy was ever confirmed (either pre-operatively or in explanted specimens)^329^. Subsequent studies have replicated these findings, and identified risk factors for wait-list drop-out as well as for disease recurrence, thus identifying potential candidates for more effective future systemic therapies^329–332^. Still, in an Irish cohort, short-term mortality (10–58 months) was observed in patients with CCA undergoing liver transplantation and receiving neoadjuvant chemoradiotherapy^330^.

The efficacy of neoadjuvant chemoradiotherapy and liver transplantation in patients with unresectable disease has led to the question of whether similar therapy should be offered to patients with resectable pCCA. The extremely limited supply of liver allografts and the need for life-long immunosuppression are important obstacles to this strategy. However, a retrospective multicentre study found that patients with unresectable pCCA undergoing combined neoadjuvant therapy plus liver transplantation had longer 5-year survival (64% versus 18%; *P* < 0.001) than patients undergoing resection who otherwise met liver transplantation criteria, and this difference remained statistically significant in an intention-to-treat analysis, even after accounting for tumour size, nodal status and PSC^333^.

The data for liver transplantation in the setting of iCCA are more preliminary than for pCCA, but might be of great value for patients with cirrhosis and tumours smaller than 2 cm. In an initial Spanish study, 5-year survival following transplantation in patients with small, incidental iCCA (<2 cm) was 65%, and this was confirmed in a larger international retrospective analysis using similar selection criteria^24,334^. In a small series of patients (*n* = 6) with very large, unresectable iCCA treated with liver transplantation after a prolonged period of disease stability following treatment with neoadjuvant chemotherapy, the 5-year survival was 83% although, importantly, recurrence was noted in 50% of the patients^335^.

#### Palliative chemotherapy

At the time of assessment of patients with CCA for palliative treatment, the following three aspects need to be considered: patient fitness as assessed in terms of ECOG-PS (patients with an ECOG-PS of ≥3 are unlikely to benefit from treatment and should be managed with best supportive care); disease distribution (patients with oligometastatic disease or with liver-only disease might be suitable for specific treatment approaches); and accessibility of tumour profiling.

Robust data support the used of first-line cisplatin and gemcitabine chemotherapy in patients with advanced disease^336,337^. The ABC-02 trial randomly assigned 410 patients with ECOG-PS ≤2 to systemic chemotherapy with gemcitabine alone or cisplatin–gemcitabine^336^; the study showed an OS benefit in favour of cisplatin–gemcitabine (HR 0.64), a benefit confirmed in the Japanese randomized phase II BT22 study^337^. Although patients with bilirubin more than twice the upper limit of normal were excluded from the ABC-02 trial, safety and feasibility data support its use in patients with a good ECOG-PS (PS 0 or 1) with jaundice who have refractory biliary obstruction due to endoluminal disease^338^. More intensive triple-chemotherapy combinations are being explored in the first-line setting, such as cisplatin–gemcitabine combined with nab-paclitaxel^339^ or with S1 (tegafur, gimeracil and oteracil)^340^, and FOLFIRINOX (5-FU, oxaliplatin and irinotecan; AMEBICA study, NCT02591030). Acelarin (NUC-1031) is a first-in-class nucleotide analogue, which, unlike gemcitabine, is independent of hENT2 (also known as SLC29A2) cellular transport and is not metabolized by cytidine deaminase, resulting in greater intracellular concentrations. Acelarin with cisplatin^341^ will be compared with gemcitabine and cisplatin combination therapy in a phase III study (NCT04163900).

After progression on first-line chemotherapy, the benefit of second-line treatment remained unclear until the past few years^342^. The phase III ABC-06 clinical trial randomly assigned 162 patients diagnosed with advanced biliary tract cancer (72% CCA) who had already progressed on first-line cisplatin–gemcitabine to active symptom control (81 patients) or active symptom control with FOLFOX (folinic acid, 5-FU and oxaliplatin) second-line chemotherapy (81 patients), with OS as the primary end-point^343^. The ABC-06 trial showed a benefit from second-line chemotherapy (adjusted HR 0.69). Although differences in median OS were modest (5.3 versus 6.2 months) between study arms, differences in survival at 6 months (35.5% versus 50.6%) and 12 months (11.4% versus 25.9%) were clinically meaningful. Based on these findings, FOLFOX can be considered a new standard of care in the second-line setting.

#### Liver-directed therapies and management of oligometastatic disease

The benchmark for liver-directed therapies was set by a subgroup analysis of the ABC trials including only those 32 patients who received cisplatin and gemcitabine for unresectable iCCA without extrahepatic metastasis^344^. The median OS in cisplatin-treated and gemcitabine-treated patients with iCCA was 16.7 months and the 3-year OS was 0%. Patients diagnosed with iCCA in liver-predominant disease might be considered for liver-directed therapies^345,346^. Options for intra-arterial therapy include transarterial radioembolization (TARE) with yttrium-90 (ref.^347^) and liver chemosaturation^232^. TARE is the most developed approach but robust evidence supporting its activity is modest^347–350^, and the randomized SIRCCA clinical trial evaluating the benefit of adding TARE to gemcitabine and cisplatin in liver-only locally advanced iCCA was prematurely interrupted because of poor recruitment (NCT02807181). Options for local therapy, in the form of external beam radiation^351^, are also available. Data are awaited from prospective studies (such as the ABC-07 study; EudraCT 2014-003656-31) to evaluate the benefit derived from such approaches in combination with systemic chemotherapy. A phase II trial including 38 patients with unresectable iCCA who received hepatic intra-arterial pump chemotherapy with floxuridine found an impressive radiological response rate of 58% and a 3-year OS exceeding 40%^352^.

#### Targeted therapies

Inhibitors of IDH1 (AG120, IDH305), IDH2 (AG221) and pan-IDH1–IDH2 (AG881) are currently being tested in patients with iCCA. AG120 (ivosidenib) was tested in 73 patients with *IDH1*-mutant advanced CCA in a phase I study^353^. The only treatment-related grade 3 or worse adverse event present in more than one patient was fatigue (two patients, 3%), and 5% had a confirmed partial response. In a preliminary phase III trial in which 185 patients with *IDH-1* mutant CCA were randomly assigned to ivosidenib or placebo^128^, ivosidenib showed a benefit in terms of progression free-survival (HR 0.37). Median OS was 10.8 months in patients receiving ivosidenib and 9.7 months in patients receiving placebo (HR 0.69); after adjustment of the OS estimation in the placebo arm for crossover (57% of patients in the placebo arm crossed over to ivosidenib at time of disease progression), the median OS in the placebo arm was 6 months. This landmark study provided level A evidence for the efficacy of targeted therapy in CCA and mandates the provision of molecular profiling in this cancer.

There have been promising preliminary data for FGFR inhibitors from phase II studies^123,354–357^. Hyperphosphataemia has been shown to be a class effect due to on-target blockade, and requires monitoring and active management^356^. Some FGFR inhibitors are currently being evaluated earlier in the disease course (first-line setting; for example, the FIGHT-302 study (NCT03656536), and the PROOF study (NCT03773302)). Furthermore, some pan-tumour studies including patients with neurotrophic RTK fusions (TRK inhibitors)^358,359^ or WNT pathway alterations such as *RNF43* mutations (porcupine inhibitors; NCT03447470)^118^ are relevant to CCA, but only for a very small percentage of patients.

#### Role of immunotherapy

To date, the clinical data on immune-directed therapies in CCA are limited. Immunotherapy approaches such as vaccines have been tested in CCA without notable success^360^. Early data are also available for CAR T cell immunotherapy^361^. In some patients, immune checkpoint blockade with monoclonal antibodies has shown remarkable and durable response rates in a variety of human malignancies^362^. Checkpoint inhibitors were shown to be effective in patients with MMR-deficient tumours (including some patients with CCA) achieving objective responses in up to 40% of patients^363^. The KEYNOTE-028 basket trial of pembrolizumab included patients with advanced biliary tract cancer. The objective response rate in this subset was 17% (4 of 23) with a median progression-free survival of 1.8 months^364^. However, the KEYNOTE-158 trial failed to confirm such activity in biliary tract tumours, with only 6% of patients responding, with a median progression-free survival of 2 months (NCT02628067) according to a preliminary report^230^. The probable way forward for the development of immunotherapy in CCA (in tumours without MMR deficiency, which respond better) requires either combination immunotherapeutic approaches targeting both the innate and adaptive immune system and/or combined strategies involving chemotherapy or radiation, already planned as part of some of the ongoing clinical trials (ABC-09 trial; NCT03260712). Overall, recommendations for CCA management are summarized in Box 1.

Box 1 Recommendations for cholangiocarcinoma management

- Surgical resection (based on the TNM criteria) is currently a potential curative option for cholangiocarcinoma (CCA).
- Adjuvant chemotherapy with capecitabine for 6 months after surgical resection with curative intent is recommended for intrahepatic CCA.
- Liver transplantation is a potentially curative option for intrahepatic and perihilar CCA; promising results in terms of overall survival have been reported and it must be considered for patients with cirrhosis and intrahepatic CCA tumours ≤2 cm.
- Combination of cisplatin and gemcitabine is the standard of care for patients with unresected tumours, as a palliative treatment.
- FOLFOX (folinic acid, fluorouracil and oxaliplatin) can be recommended as second-line standard of care chemotherapy.
- Molecular profiling of cancer tumour tissue is highly recommended because it could provide access to effective, personalized, treatment options; phase III trials with IDH1–IDH2 or FGFR inhibitors as first- and/or second-line treatment are ongoing.

### Mechanisms of chemoresistance

A major limitation in the management of patients with CCA is the lack of response to pharmacological treatment. Mechanisms of chemoresistance (MOC) accounting for the marked multidrug resistance phenotype of CCA are still poorly understood^365^. Nevertheless, to identify the so-called resistome, including a set of proteins involved in the lack of response to chemotherapies, is required to predict treatment failure and to adapt the therapeutic strategy to the evolving defences of the tumour^366^. Genes involved in MOC are expressed by normal cholangiocytes, with different roles in their physiology, such as protection against potentially harmful compounds present in bile. Their expression during carcinogenesis accounts for intrinsic chemoresistance, whereas upregulation in response to treatment contributes to acquired chemoresistance^365,367^.

**Fig. 8:**
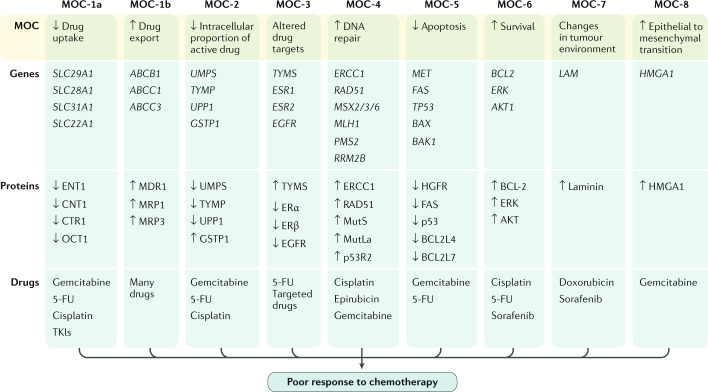
Mechanisms of chemoresistance in cholangiocarcinoma. Relevant genes and proteins involved in each type of mechanism of chemoresistance (MOC-1 to MOC-7) in cholangiocarcinoma (CCA) are shown, either because they are upregulated or downregulated or their function is enhanced or impaired. Drugs whose efficacy is affected by these changes in the resistome are shown. 5-FU, 5-fluorouracil; TKI, tyrosine-kinase inhibitor.

On the basis of their mechanism of action, MOC genes have been classified into seven groups^365,366^ (Fig. 8). For instance, impaired expression and/or function of plasma membrane solute carriers involved in drug uptake (MOC-1a) results in decreased sensitivity to drugs that cannot reach their intracellular targets. Downregulation of concentrative nucleoside transporters and equilibrative nucleoside transporters, involved in the uptake of nucleoside analogues, such as gemcitabine and 5-FU, or the copper transporter CTR1, involved in cisplatin uptake, lead to reduced sensitivity of CCA cells to these drugs^368,369^. OCT1 downregulation in CCA^369–371^ is involved in the lack of response to sorafenib^372^. ATP-binding cassette pumps are important factors accounting for the low intracellular concentrations of anticancer drugs (MOC-1b). The multidrug resistance protein 1, able to export etoposide, doxorubicin, paclitaxel and vinblastine, has been detected in gallbladder epithelium^373^, whereas the multidrug resistance-associated proteins MRP1 and MRP3 have been observed in CCA^369^. MRP1 expression has been associated with poor prognosis of patients with iCCA^374^. Changes in metabolic enzymes can reduce the proportion of active drugs inside tumour cells (MOC-2). Uridine monophosphate synthase, thymidine phosphorylase and uridine phosphorylase 1, which transforms 5-FU and gemcitabine into their active metabolites, are upregulated in many 5-FU-sensitive CCA tumours^375^. Glutathione *S-*transferase P, which inactivates drugs, such as cisplatin, by conjugation with glutathione, is highly expressed in CCA^376^.

Importantly, the response to anticancer agents is dependent on the expression and/or function of their molecular targets (MOC-3). High expression of thymidylate synthase in human biliary tract carcinoma cells has been related to insensitivity to 5-FU^377^. Studies in CCA cells expressing oestrogen receptors have suggested that selective agonists could be a therapeutic option in patients with CCA^378^. Expression levels of EGFR have been associated with the sensitivity of CCA cells to targeted agents^379^. Moreover, increased ability of tumour cells to repair drug-induced DNA damage (MOC-4) can also contribute to chemoresistance. The endonuclease DNA excision repair protein 1 removes bulky DNA adducts, whose levels have been associated with the response to cisplatin in patients with CCA^380^. Changes in promoter methylation of protein complexes involved in DNA MMR, such as human MLH1, affect prognosis in CCA^381^. Upregulation of ribonucleotide reductase p53R2 has been proposed as a predictive marker of CCA resistance to gemcitabine^382^. Decreased expression and/or function of pro-apoptotic proteins results in reduced efficacy of chemotherapy (MOC-5a). Downregulation of NK4 in response to 5-FU treatment induces resistance in CCA cells to this drug in vitro^55^. Downregulation of *BAX*, *BAK*, caspase 3 and caspase 9 has been associated with drug resistance in cancer^383^. Interaction of Fas cell surface death receptor with calmodulin inhibits Fas-induced apoptosis and results in CCA chemoresistance^384^. By contrast, enhanced expression and/or function of anti-apoptotic proteins also reduces the efficacy of chemotherapy (MOC-5b). Overexpression of ERK and BCL-2 or the overactivation of the PI3K–AKT and RAF–MEK–ERK pathways have been associated with chemoresistance in CCA cells^385^. CCA usually shows lower vascularity than in HCC — probably as a result of its extensive stroma — which is associated with greater malignant potential^386^. Moreover, this reduced vascularity due to extensive stroma can limit the access of administered drugs to all tumour cells (MOC-7). This feature and other characteristics that affect the TME, including hypoxia and reduced pH, could reduce the effectiveness of anticancer drugs (MOC-6)^387^. It has been proposed that ECM proteins, such as laminin-332, induce resistance to doxorubicin and sorafenib in CCA^197^. Finally, activation of EMT is also involved in resistance to chemotherapy (MOC-7)^366^. High mobility group A1 protein promoted CCA tumorigenicity and conferred resistance to gemcitabine in vitro in CCA cell lines^388^.

## Future directions and recommendations

The known risk factors for CCA are only involved in ∼20% of cases, indicating the urgent need to ascertain other causes of disease to improve awareness and screening policies for early diagnosis, which might substantially influence patient outcomes. However, considering some established risk factors, potential prevention strategies and lifestyle-modifying concerted actions should be developed to increase awareness. For instance, HBV vaccination, improvements in treatment of HBV infection, HCV infection and nonalcoholic fatty liver disease, and promotion of specific campaigns aiming to reduce alcohol and tobacco consumption, as well as obesity, might markedly influence both the incidence and mortality of CCA.

The accurate recording of epidemiological data (incidence and mortality of each subtype of CCA) and the elucidation of the environmental risk factors and their interplay with genetic and molecular determinants in cholangiocarcinogenesis are extremely important. In this regard, a new coding system was recently approved (ICD-11 and ICD-O-4)^20^ that better reflects the CCA subtypes — intrahepatic, perihilar and extrahepatic (distal) CCAs — and will start to be used in 2021. Importantly, diagnostic data need to be recorded uniformly and accurately by clinicians, administrators and cancer registries. Awareness of the historical miscoding of CCA should be raised amongst all members of the multidisciplinary team, and at all levels. We need to ensure the appropriate education of coding personnel, who should have senior clinician input to check the accuracy of coding data. Furthermore, accuracy of coding data should be regularly audited. In the future, given advances in our understanding of the genetic drivers for subtypes of CCA, perhaps coding of CCA might also involve molecular profiling.

CCAs are highly heterogeneous at both the intertumoural and intratumoural levels, and have a very poor prognosis. The high heterogeneity and chemoresistance of CCAs represent a limitation for common therapeutic strategies, but it is a unique opportunity for personalized, targeted therapies. Up to 50% of CCAs have current druggable mutations, amplifications or fusions (for example, *IDH1*, *IDH2*, *BRAF*, *FGFR*, *HER2*, *PIK3CA*, *MET*, among others), opening a new opportunity for therapeutic intervention that deserves intense basic and clinical research. In fact, targeting these mutations is amenable and is already a reality in other types of cancer^389,390^. In this scenario, the treatment of patients with breast cancer or colorectal cancer with trastuzumab (anti-HER2)^391^ and cetuximab–panitumumab (anti-EGFR)^392^ is an example of the successful use of targeted therapy. Therefore, exploration of targeted therapies on a background of standard of care chemotherapy should be continued for CCA. In addition, combined efforts should be made to develop curative therapies. Cost is a major drawback and funding opportunities should be revisited and improved, in parallel with increased awareness amongst the research community, general society, funding agencies and the pharmaceutical industry. Concerted action aimed at increasing the cooperation of these entities should be realized to achieve new effective therapies.

Interactions between cancer cells, CSCs and the TME, as well as the evident clonal evolution and cellular aberrations (genomic, genetic, epigenetic and molecular) contribute to CCA heterogeneity. New technical approaches such as single-cell RNA or DNA sequencing could provide novel critical information about cellular heterogeneity, in both the tumour compartment and the stroma, by capturing genomic and/or genetic alteration with a resolution at the level of the single cell.

New classifications of CCAs based on the combination of clinical, radiological, histological, genomic and molecular features, and later evaluation of their associations with prognosis and treatment response, are mandatory. In addition, it is fundamental to include the resistome in this equation, since the baseline and acquired MOCs will undoubtedly contribute to the success of the therapies tested. The potential determination of the resistome in liquid biopsies (that is, in cfDNA) would open a new avenue for personalized treatment. Future clinical trials should consider the stratification of patients considering clinicopathological subtyping and risk factors, as well as the genomic landscape. Moreover, patient selection for surgery, local therapies, chemotherapy and targeted therapies must be improved. Similar to the tumour compartment, a better stratification of these alterations within the microenvironment could help in the design of innovative treatment options including immunotherapies (such as immune checkpoints and CAR T cell therapy) and ECM-oriented treatments. The majority of clinical trials performed so far for advanced CCA did not take all these considerations into account, which might explain, at least in part, the disappointing results obtained. Thus, action needs to be taken to bring together experts across different fields. In addition, robust circulating biomarkers are needed for the accurate diagnosis of CCA, as well as to predict prognosis and treatment response. Non-coding RNA, specifically miRNAs, long non-coding RNAs and circular RNAs^393,394^ and circulating proteins and/or metabolites could represent such promising biomarkers, due to their easy detection and stability in biological fluids, either free or encapsulated into extracellular vesicles. In this regard, international collaborative projects, such as the ESCALON project (competitively funded by the European Union as part of the Horizon 2020 programme), are warranted to improve the understanding, prediction (risk factors) and diagnosis (biomarkers) of CCA not only in Europe and North America, but also in Latin America and other continents.

CCA management nowadays requires dedicated centres with multidisciplinary expertise that enable the proper translation of basic investigations to clinical practice. International collaborative networks of multidisciplinary scientists such as the ENS-CCA are especially important as they are accelerating acquisition of scientific knowledge on this cancer, which then influences clinical practice. In particular, it is important to highlight the ENS-CCA Action EURO-CHOLANGIO-NET, a European Horizon 2020 competitive programme (2019–2023) that has the objective to create and boost multidisciplinary and cross-sectional studies to decipher the biological jigsaw of CCA. This open, structured initiative has received the support of the European Commission and is endorsed by the European Association for the Study of the Liver, the International Liver Foundation, several research and development companies, and the CCA patient associations (including the Cholangiocarcinoma Foundation in the USA and the Alan Morement Memorial Fund (AMMF) in the UK). During the coming years, EURO-CHOLANGIO-NET will concentrate in the following objectives: shorten the current gaps in CCA knowledge and applications by overcoming the limitation of the small number of cases through the development of international clinical, histological and radiological registries, which are necessary to dissect the multilevel heterogeneity of CCAs; improve translation by generating consensus on appropriate experimental models of CCA, diagnostic and/or prognostic biomarkers and imaging techniques, and clinical management; dissect intertumoural and intratumoural heterogeneity to define specific features for early diagnosis of each CCA subtype; rationalize cost-efficient, personalized, targeted therapies for CCA by defining the driver mutations, epigenetic alterations, and transcriptome of each CCA histomorphological subtype; and develop novel drugs and therapeutic strategies. The ENS-CCA, together with dedicated foundations such as the AMMF and the Cholangiocarcinoma Foundation, as well as other international networks and collaborators have contributed to the creation of the Global Cholangiocarcinoma Alliance, which has the aim of joining forces to increase awareness of this cancer and to establish a global voice in CCA through community collaborations. In Asia, an important consortium was created — the Thailand Initiative in Genomics and Expression Research for Liver Cancer (TIGER-LC) — to identify genomic and endemic factors that could modify CCA and HCC susceptibility and progression^395^. Combining the efforts from experts worldwide will definitely contribute to improved understanding of CCA and will reinforce the necessary link between basic and clinical science, and therefore hopefully improve patient welfare.

## Conclusions

CCAs are highly aggressive and heterogeneous, at both the intertumoural and intratumoural levels, resulting in poor prognosis. Different risk factors, interactions between cancer cells, CSCs and the TME, as well as the evident clonal evolution and genetic and/or epigenetic aberrations contribute to CCA heterogeneity. Different CCA molecular subtypes, with distinct prognoses and responses to therapy, have already been described. Tumour resection is still the only potentially curative option for these patients, although only a small percentage of patients are eligible and the percentage recurrence is high. We still lack of accurate noninvasive biomarkers for the diagnosis and to estimate the prognosis in patients with CCA. Furthermore, knowledge of the MOCs of these cancers needs to be expanded, but current information should be included in the future treatment decisions. Although important information has already been unveiled, CCA is still an open field of research, with important gaps that need to be filled (Table 4). Therefore, all the efforts must be gathered to try to go beyond and decipher the complexity of CCA.

**Table 4:** Research priorities for cholangiocarcinoma

Category	Priority	Timescale	Cost–benefit ratio	Initiative
***Basic or translational research***				
Expertise	Dedicated centres with multidisciplinary expertise are urgently required	Long-term	Proper translation of basic investigation to clinical practice and amelioration of CCA management will be boosted	NA
Expertise	Dedicated special topic conferences bringing together basic and clinical researchers, industry and also stakeholders and governmental counterparts must be implemented	Short-term	This constitutes a great opportunity to share fundamental research findings, develop multi-team international collaborations and also engage political institutions to speed up the translation of research into clinics	ENS-CCA has established a biannual meeting; CCF and AMMF have annual meetings; EASL has an annual meeting on liver cancer
Genetics	GWAS in CCA are still missing	Short-term	The identification of specific SNPs that might be related to CCA development might be of great help in identifying patients with early disease	An International GWAS is currently ongoing with the support of the CCF; future genomic DNA samples will be needed for a validation phase, particularly from less-represented and developing countries
Biomarkers	International validation studies of biomarkers for CCA are mandatory to translate the preliminary results available from multi-omic studies into clinical practice through evidence-based recommendations; these studies should include large cohorts of patients with histologically proven diagnosis, appropriate control groups (e.g. cirrhotic, non-cirrhotic, HBV and HCV infection, steatosis), and further prospective validation in the setting of clinical trials	Medium- or long-term	The identification of new diagnostic and prognostic biomarkers will greatly influence health systems, enabling the early identification of patients and treatment responses, and will be of value for follow-up after surgery	An international project for the validation of diagnostic biomarkers for hepatobiliary cancers is ongoing (ESCALON; European H2020, SC1-BHC-18-2018, €3.3 million)
Platforms	Platforms that facilitate translational research, enabling access to tissue and blood samples from patients enrolled in clinical trials and also treated with standard of care therapies are required	Medium- or long-term	A better understanding of primary and acquired resistance to systemic therapies together with other predictor factors of response will be possible and will enable better therapeutic decisions	NA
Animal models	Different animal models of CCA have been proposed in the past few years; however, deep characterization of their histomorphology, pathobiology, cells of origin, and genomic, epigenetic and molecular features are still missing, as well as analysis of their similarities to and differences from the different CCA human subtypes	Medium- or long-term	The consequences of using incorrect animal models might provide false-positive results that will probably lead to failure of translation into the clinic, and also false-negative’ results that will result in potential missed opportunities for new therapies; appropriate CCA animal models will enable proper translation of new drugs into the clinic	International collaborative efforts are currently being coordinated within ENS-CCA (European COST Action EURO-CHOLANGIO-NET, CA18122) to develop strict guidelines and define the distinct models of CCA with respect to human subtype counterparts
Animal models	Experimental models of CCA under chronic liver damage (e.g. chronic cholestasis, PSC, cirrhosis, HCV, HBV, steatosis) are needed, which could recapitulate better the disease origin and progression and that reproduce the standards of clinical care (e.g. surgical resection of desmoplastic liver tumour followed by adjuvant chemotherapy of metastatic disease).	Medium or long-term	Appropriate CCA animal models will enable the proper translation of new drugs into clinics	NA
Animal models	The capacity of xenobiotics to promote cholangiocarcinogenesis should be tested in vivo to identify potential risk factors linked to the exposome, and to help in the development of new animal models of CCA	Medium- or long-term	Appropriate CCA animal models will enable the proper translation of new drugs into the clinic	NA
Immunotherapy	The value of immunotherapy and CAR T cell immunotherapy on the different CCA subtypes needs to be determined	Medium- or long-term	Applying immunotherapy in CCA treatment might substantially improve patient outcomes and quality of life	NA
Molecular and biological characterization of CCA tumours	Molecular and biological aspects of CCA tumours must be analysed, and the involvement of the desmoplastic stroma in such mechanisms and their link to treatment response must be implemented in clinical practice; MOCs should be explored in the setting of translational research networks linked to the ongoing clinical trials in CCA to derive a better understanding of the resistance mechanisms of both current treatment strategies and those under development	Medium- or long-term	Understanding the MOC in CCA treatment might help in the development of new personalized therapeutic strategies to overcome this drawback	NA
Characterization of iCCA subtypes	Combined morphological and molecular description of intrahepatic CCA subtypes is urgently needed	Long-term	It will be possible to elucidate the aetiology and actionable molecular alterations in intrahepatic CCA	NA
TME	An in-depth analysis of the TME is needed, including the role of cancer-associated fibroblasts, innate and adaptive immune cells and extracellular biomatrix	Long-term	A better understanding of carcinogenesis and therapeutic dependencies will enable the development of new and more effective drugs	NA
***Clinical research***				
Awareness	Awareness actions and prevention strategies, mainly related to CCA risk factors (HCV and HBV infection, obesity, NAFLD and/or NASH, alcohol consumption, tobacco use, liver fluke infestation) should be highly promoted worldwide	Short-term	With increased awareness of potential CCA risk factors, tumour incidence and prevalence might markedly drop	The Global CCA Alliance shares this vision and mission
Adjuvancy	Further development of adjuvant strategies is required	Short-term	It will be possible to improve outcomes of patients with resectable disease and to reduce the risk of tumour recurrence	NA
First-line treatments	An understanding of he role of first-line triple-chemotherapy combinations in the setting of advanced CCA is needed and will require randomized clinical trials comparing such strategies with the current standard of care (cisplatin–gemcitabine)	Medium- or long-term	It will be possible to improve patient treatment and outcomes and better decide which drugs should be used to treat patients	NA
Local therapies	The role of local therapies such as liver transplantation, liver embolization, liver chemosaturation and external beam radiation therapy should be explored in the setting of prospective clinical trials	Medium- or long-term	It will be possible to improve patient treatment and outcomes and better decide which drugs should be used to treat patients	NA
Second-line treatments	Further research is required to improve second-line systemic treatment strategies in CCA	Long-term	It will be possible to maximize benefit to patients	NA
Collection and processing of samples	Standard guidelines for the collection of CCA tumour tissue, serum and other biological samples, and also for sample processing and acquisition of clinical data should be developed	Short- or medium-term	Variability between centres and countries/regions will be greatly reduced, resulting in more robust data	ENS-CCA
Staging	Currently available staging classifications (AJCC Cancer Staging; TNM) need to be reviewed to reflect the emerging prognostic factors	Medium- or long-term	Better classification and stratification of patients will greatly aid in deciding on the therapeutic regimen and which patients should undergo curative resection and/or liver transplantation	NA
Risk factors	Identification of CCA risk factors is of great interest	Long-term	Enhancing and implementing screening policies that would enable the early diagnosis of CCA at stages when curative surgery is possible	NA
Machine learning	The field of machine learning and development of algorithms and statistical models should be explored	Medium- or long-term	Might gather all the information necessary for CCA diagnosis, prognosis prediction and therapeutic decisions, in shorter periods of time and with more reproducibility and accuracy	EU funding calls dedicated to this field
AI	The field of AI for the compilation of multi-omic data to improve personalized medicine should be evaluated	Medium- or long-term	Development and testing AI technologies on multi-omics and health data repositories; identifying new knowledge; supporting clinical research and decision making	EU funding calls dedicated to this field

AI, artificial intelligence; CCA, cholangiocarcinoma; CCF, Cholangiocarcinoma Foundation; EASL, European Association for the Study of Liver Disease; ENS-CCA, European Network for the Study of Cholangiocarcinoma; GWAS, genome-wide association study; HBV, hepatitis B virus; HCV, hepatitis C virus; MOC, mechanism of chemoresistance; NA, not available; NAFLD, nonalcoholic fatty liver disease; NASH, nonalcoholic steatohepatitis; PSC, primary sclerosing cholangitis; SNP, single nucleotide polymorphism.

### Supplementary information

Supplementary information
