# Supplementary material for: Cholangiocarcinoma 2020: the next horizon in mechanisms and management
Source: Nat Rev Gastroenterol Hepatol. 2020 Jun 30;17(9):557–88. doi: 10.1038/s41575-020-0310-z (PMC7447603; doi:10.1038/s41575-020-0310-z)
Supplement: Supplementary file 1 — Supplementary information [file 41575_2020_310_MOESM1_ESM.pdf]

Cholangiocarcinoma 2020: the next horizon in mechanisms and management

In the format provided by the authors and unedited

Supplementary information

Supplementary Figure 1 | Diagnostic and prognostic biomarkers for CCA. Schematic representation of potential biomarkers (i.e. in extracellular vesicles, proteins/cytokines, metabolites and microRNAs) found in serum, bile, urine and tumour tissue with diagnostic and/or prognostic capacity. AUC, area under the curve; CCA, cholangiocarcinoma; EV, extracellular vesicle.

EV cargo[S1]	Levels	Comparison	AUC
AMPN	Up	CCA (n=43) *vs* healthy controls (n=32)	0.878
VNN1	Up	CCA (n=43) *vs* healthy controls (n=32)	0.876
PIGR	Up	CCA (n=43) *vs* healthy controls (n=32)	0.844
FCN2	Up	Early stage CCA (n=13) *vs* PSC (n=30)	0.956
ITIH4	Up	Early stage CCA (n=13) *vs* PSC (n=30)	0.881
FIBG	Up	Early stage CCA (n=13) *vs* PSC (n=30)	0.881
FIBG	Up	iCCA (n=12) *vs* HCC (n=29)	0.894
A1AG1	Up	iCCA (n=12) *vs* HCC (n=29)	0.845
VTDB	Up	iCCA (n=12) *vs* HCC (n=29)	0.823

Protein/ Cytokine	Levels	Comparison	AUC
MMP7[S2]	Up	CCA (n=44) *vs* benign biliary tract disease (n=36)	0.730
Osteopontin[S3]	Up	CCA (n=80) *vs* healthy controls (n=42)	0.964
IL-6[S4]	Up	Bile duct cancer (n=26) *vs* healthy controls (n=23)	0.875
S100A6[S5]	Up	CCA (n=29) *vs* healthy controls (n=22)	0.909
DKK1[S6]	Up	iCCA (n=37) *vs* healthy controls (n=50)	0.872
SSP411[S7]	Up	CCA (n=35) *vs* "cholangitis (n=13) and healthy controls (n=23)"	0.913

Metabolite[S8]	Levels	Comparison	AUC
SM(43:2)<br>PC(O-16:0/20:3)<br>PC(O-18:0/18:2)<br>SM(d18:2/16:0)<br>Cer(d18:1/16:0)<br>SM(42:3)	Up<br>Down<br>Down<br>Up<br>Up<br>Up	iCCA (n=35) *vs* HCC (n=34)<br>Biopsy proven	DIS:0.900<br>VAL: 0.981
PC(34:3)<br>Histidine	Down	iCCA (n=35) *vs* PSC (n=35)<br>Biopsy proven	DIS: 0.990<br>VAL: 0.995

Metabolite	Levels	Comparison	AUC
Phosphatidylcholine<br>Bile acids<br>Cholesterol/lipid[S9]	Down	CCA (n=16) *vs*<br>begin non-PSC biliary diseases (n=27)	SEN: 88.9%<br>SPE: 87.1%
Glycine-conjugated bile acids<br>Phosphatidylcholines[S10]	Up<br>Down	"Inoperable pCCA (n=3) and dCCA (n=2)" *vs* non-malignant biliary diseases without cholestasis (n=20)	SEN: 80%<br>SPE: 95%

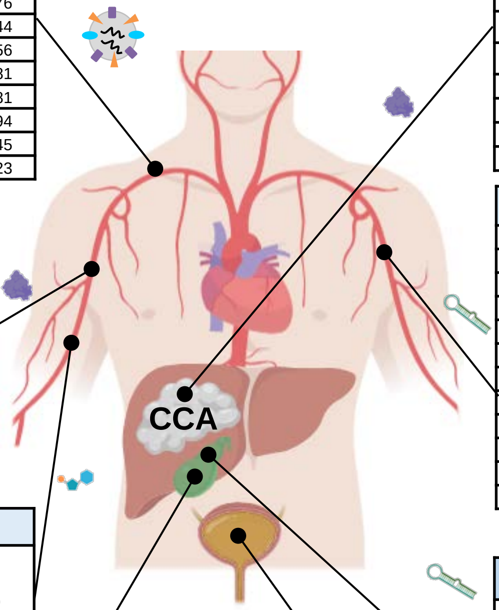

Gene	Expression	Overall survival	Recurrence-free survival
KRAS[S11]	High	Low	Low
TP53[S11]	Low	Low	Low
FASCIN/FSCN1[S12]	High	Low	-
EGFR[S13]	High	Low	-
MUC1[S14]	High	Low	-
MUC4[S15]	High	Low	-
CDKN1B/p27[S16]	Low	Low	-
MiR21[S17,S18]	High	Low	Low

miRNA	Levels	Comparison	AUC
miR-21[S17]	Up	iCCA (n=74) *vs* healthy controls (n=74)	0.908
miR-192[S19]	Up	CCA (n=11) *vs* healthy controls (n=9)	0.809
miR-26a[S20]	Up	CCA (n=66) *vs* healthy controls (n=66)	0.899
miR-150[S21]	Up	iCCA (n=15) *vs* healthy controls (n=15)	0.791
miR-194 & miR-483-5p[S22]	Up	CCA (n=30) *vs* healthy controls (n=30)	0.810
miR-222 & miR-483-5p[S22]	Up	CCA (n=30) *vs* PSC (n=30)	0.770
miR-126[S22]	Down	CCA (n=30) *vs* PSC (n=30)	0.870
miR-26a[S22]	Down	CCA (n=30) *vs* PSC (n=30)	0.780
miR-30b[S22]	Down	CCA (n=30) *vs* PSC (n=30)	0.780
miR-122[S22]	Down	CCA (n=30) *vs* PSC (n=30)	0.650
miR-106a[S23]	Down	CCA (n=103) *vs* healthy controls (n=20)	0.890
miR-1281[S24]	Down	CCA (n=31) *vs* PSC (n=40)	0.830

miRNA	Levels	Comparison	AUC
miR-640[S24]	Up	PSC-CCA (n=12) *vs* PSC (n=52)	0.810
miR-412[S24]	Up	PSC-CCA (n=12) *vs* PSC (n=52)	0.810
miR-1537[S24]	Up	PSC-CCA (n=12) *vs* PSC (n=52)	0.780
miR-3189[S24]	Up	PSC-CCA (n=12) *vs* PSC (n=52)	0.800
miR-9[S25]	Up	Biliary tract cancer (n=9) *vs* choledocholithiasis (n=9)	0.975
miR-145[S25]	Up	Biliary tract cancer (n=9) *vs* choledocholithiasis (n=9)	0.975

miRNA	Levels	Comparison	AUC
miR-21 & miR-192[S26]	Up	CCA (n=22) *vs* helathy controls (n=21)	0.849

Supplementary Table 1 |Selected ongoing and completed clinical trials of therapeutic agents for cholangiocarcinoma

Clinical trial name	Study arms	*n*	Primary endpoint	Secondary endpoint
BCAT	Observation alone vs gemcitabine	225 (all extrahepatic CCA)	Overall survival; no differences	No benefit in RFS; no differences based on lymph node and margin status
PRODIGE-12	Observation alone vs gemcitabine and oxaliplatin	186 (156 CCA)	RFS and HRQOL: no differences in RFS or time to deterioration of HRQOL	Overall survival was not different between arms
BILCAP	Observation alone vs capecitabine	447 (368 CCA)	Overall survival; benefit showed in the pre-specified sensitivity analysis (HR, 0.71; P = 0.01)	RFS HR 0.75, P = 0.033
ACTICCA-1 (NCT02170090)	Observation alone vs cisplatin and gemcitabine	Target: 781; ongoing	Disease-free survival at 24 months	—

CCA, cholangiocarcinoma; HRQOL, health-related quality of life; HR, hazard ratio; RFS, relapse-free survival.

Supplementary References

S1 Arbelaiz, A. *et al.* Serum extracellular vesicles contain protein biomarkers for primary sclerosing cholangitis and cholangiocarcinoma. *Hepatology* **66**, 1125-1143, doi:10.1002/hep.29291 (2017).

S2 Leelawat, K., Sakchinabut, S., Narong, S. & Wannaprasert, J. Detection of serum MMP-7 and MMP-9 in cholangiocarcinoma patients: evaluation of diagnostic accuracy. *BMC Gastroenterol* **9**, 30, doi:10.1186/1471-230X-9-30 (2009).

S3 Loosen, S. H. *et al.* Elevated levels of circulating osteopontin are associated with a poor survival after resection of cholangiocarcinoma. *J Hepatol* **67**, 749-757, doi:10.1016/j.jhep.2017.06.020 (2017).

S4 Cheon, Y. K. *et al.* Diagnostic utility of interleukin-6 (IL-6) for primary bile duct cancer and changes in serum IL-6 levels following photodynamic therapy. *Am J Gastroenterol* **102**, 2164-2170, doi:10.1111/j.1572-0241.2007.01403.x (2007).

S5 Onsurathum, S. *et al.* Proteomics detection of S100A6 in tumor tissue interstitial fluid and evaluation of its potential as a biomarker of cholangiocarcinoma. *Tumour Biol* **40**, 1010428318767195, doi:10.1177/1010428318767195 (2018).

S6 Shi, R. Y. *et al.* High expression of Dickkopf-related protein 1 is related to lymphatic metastasis and indicates poor prognosis in intrahepatic cholangiocarcinoma patients after surgery. *Cancer* **119**, 993-1003, doi:10.1002/cncr.27788 (2013).

S7 Shen, J. *et al.* Comparative proteomic profiling of human bile reveals SSP411 as a novel biomarker of cholangiocarcinoma. *PLoS One* **7**, e47476, doi:10.1371/journal.pone.0047476 (2012).

S8 Banales, J. M. *et al.* Serum Metabolites as Diagnostic Biomarkers for Cholangiocarcinoma, Hepatocellular Carcinoma, and Primary Sclerosing Cholangitis. *Hepatology*, doi:10.1002/hep.30319 (2018).

S9 Albiin, N. *et al.* Detection of cholangiocarcinoma with magnetic resonance spectroscopy of bile in patients with and without primary sclerosing cholangitis. *Acta Radiol* **49**, 855-862, doi:10.1080/02841850802220092 (2008).

S10 Sharif, A. W. *et al.* Metabolic profiling of bile in cholangiocarcinoma using in vitro magnetic resonance spectroscopy. *HPB (Oxford)* **12**, 396-402, doi:10.1111/j.1477-2574.2010.00185.x (2010).

S11 Nepal, C. *et al.* Genomic perturbations reveal distinct regulatory networks in intrahepatic cholangiocarcinoma. *Hepatology* **68**, 949-963, doi:10.1002/hep.29764 (2018).

S12 Mao, X. et al. Differential expression of fascin, E-cadherin and vimentin: Proteins associated with survival of cholangiocarcinoma patients. *Am J Med Sci* **346**, 261-268, doi:10.1097/MAJ.0b013e3182707108 (2013).

S13 Yoshikawa, D. et al. Clinicopathological and prognostic significance of EGFR, VEGF, and HER2 expression in cholangiocarcinoma. *Br J Cancer* **98**, 418-425, doi:10.1038/sj.bjc.6604129 (2008).

S14 Boonla, C. et al. MUC1 and MUC5AC mucin expression in liver fluke-associated intrahepatic cholangiocarcinoma. *World J Gastroenterol* **11**, 4939-4946, doi:10.3748/wjg.v11.i32.4939 (2005).

S15 Shibahara, H. et al. MUC4 is a novel prognostic factor of intrahepatic cholangiocarcinoma-mass forming type. *Hepatology* **39**, 220-229, doi:10.1002/hep.20031 (2004).

S16 Fiorentino, M. et al. Low p27 expression is an independent predictor of survival for patients with either hilar or peripheral intrahepatic cholangiocarcinoma. *Clin Cancer Res* **7**, 3994-3999 (2001).

S17 Wang, L. J. *et al.* MiR-21 promotes intrahepatic cholangiocarcinoma proliferation and growth in vitro and in vivo by targeting PTPN14 and PTEN. *Oncotarget* **6**, 5932-5946, doi:10.18632/oncotarget.3465 (2015).

S18 Chusorn, P. *et al.* Overexpression of microRNA-21 regulating PDCD4 during tumorigenesis of liver fluke-associated cholangiocarcinoma contributes to tumor growth and metastasis. *Tumour Biol* **34**, 1579-1588, doi:10.1007/s13277-013-0688-0 (2013).

S19 Silakit, R. *et al.* Circulating miR-192 in liver fluke-associated cholangiocarcinoma patients: a prospective prognostic indicator. *J Hepatobiliary Pancreat Sci* **21**, 864-872, doi:10.1002/jhbp.145 (2014).

S20 Wang, L. J. *et al.* Serum miR-26a as a diagnostic and prognostic biomarker in cholangiocarcinoma. *Oncotarget* **6**, 18631-18640, doi:10.18632/oncotarget.4072 (2015).

S21 Wang, S. *et al.* Upregulated circulating miR-150 is associated with the risk of intrahepatic cholangiocarcinoma. *Oncol Rep* **33**, 819-825, doi:10.3892/or.2014.3641 (2015).

S22 Bernuzzi, F. *et al.* Serum microRNAs as novel biomarkers for primary sclerosing cholangitis and cholangiocarcinoma. *Clin Exp Immunol* **185**, 61-71, doi:10.1111/cei.12776 (2016).

S23 Cheng, Q. *et al.* Circulating miR-106a is a Novel Prognostic and Lymph Node Metastasis Indicator for Cholangiocarcinoma. *Sci Rep* **5**, 16103, doi:10.1038/srep16103 (2015).

S24 Voigtlander, T. *et al.* MicroRNAs in Serum and Bile of Patients with Primary Sclerosing Cholangitis and/or Cholangiocarcinoma. *PLoS One* **10**, e0139305, doi:10.1371/journal.pone.0139305 (2015).

S25 Shigehara, K. *et al.* Real-time PCR-based analysis of the human bile microRNAome identifies miR-9 as a potential diagnostic biomarker for biliary tract cancer. *PLoS One* **6**, e23584, doi:10.1371/journal.pone.0023584 (2011).

S26 Silakit, R. et al. Urinary microRNA-192 and microRNA-21 as potential indicators for liver fluke-associated cholangiocarcinoma risk group. *Parasitol Int* **66**, 479-485, doi:10.1016/j.parint.2015.10.001 (2017).
